# Phenotypical and functional heterogeneity of neural stem cells in the aged hippocampus

**DOI:** 10.1111/acel.12958

**Published:** 2019-04-15

**Authors:** Soraya Martín‐Suárez, Jorge Valero, Teresa Muro‐García, Juan Manuel Encinas

**Affiliations:** ^1^ Achucarro Basque Center for Neuroscience Leioa Spain; ^2^ The Basque Foundation for Science IKERBASQUE Bilbao Spain; ^3^ University of the Basque Country (UPV/EHU) Leioa Spain

**Keywords:** aging, adult neurogenesis, hippocampus, neural stem cells

## Abstract

Adult neurogenesis persists in the hippocampus of most mammal species during postnatal and adult life, including humans, although it declines markedly with age. The mechanisms driving the age‐dependent decline of hippocampal neurogenesis are yet not fully understood. The progressive loss of neural stem cells (NSCs) is a main factor, but the true neurogenic output depends initially on the actual number of activated NSCs in each given time point. Because the fraction of activated NSCs remains constant relative to the total population, the real number of activated NSCs declines in parallel to the total NSC pool. We investigated aging‐associated changes in NSCs and found that there are at least two distinct populations of NSCs. An alpha type, which maintains the classic type‐1 radial morphology and accounts for most of the overall NSC mitotic activity; and an omega type characterized by increased reactive‐like morphological complexity and much lower probability of division even under a pro‐activation challenge. Finally, our results suggest that alpha‐type NSCs are able to transform into omega‐type cells overtime and that this phenotypic and functional change might be facilitated by the chronic inflammation associated with aging.

## 1 INTRODUCTION

In the dentate gyrus of the adult hippocampus, a resident population of radial glia‐like neural stem cells (NSCs) with astrocytic properties (Seri, García‐Verdugo, McEwen, & Alvarez‐Buylla, 2001) generates neurons throughout adulthood in most mammals including humans (Eriksson et al., 1998; Moreno‐Jiménez et al., 2019; Spalding et al., 2013). Neuronal precursors are generated from NSCs through asymmetric division and after a series of steps involving proliferation and differentiation (Kempermann, Jessberger, Steiner, & Kronenberg, 2004) integrate functionally in the hippocampal synaptic network (van Praag et al., 2002). The addition of new neurons in the hippocampus is important for new memory formation (Farioli Vecchioli, Sacchetti, Nicolis di Robilant, & Cutuli, 2018); learning (Zhang, Zou, He, Gage, & Evans, 2008); and responses to stress, anxiety, and fear (Bergami et al., 2008; Saxe et al., 2006; Snyder, Soumier, Brewer, Pickel, & Cameron, 2011), and it is necessary for the efficacy of antidepressants (Perera et al., 2011; Santarelli et al., 2003). Adult neurogenesis however declines sharply with age (Ben Abdallah, Slomianka, Vyssotski, & Lipp, 2010; Kuhn, Dickinson‐Anson, & Gage, 1996). In humans, confronting new data have been published recently suggesting that hippocampal neurogenesis declines sharply in the infant brain (Sorrells et al., 2018) but also indicating that adult neurogenesis could be maintained throughout aging (Boldrini et al., 2018; Moreno‐Jiménez et al., 2019). Aging‐related decline in adult neurogenesis is due to several mechanisms such as the loss of overall proliferative capacity (Cameron & McKay, 1999) and the progressive depletion of the NSC population (Bonaguidi et al., 2011; Encinas et al., 2011; Walter, Keiner, Witte, & Redecker, 2011). While the loss of overall proliferation is caused by the systemic and niche changes (glucocorticoid regulation, chronic inflammation, trophic factor imbalance, etc.) associated with aging, the progressive depletion of the NSC population can be mostly explained by the NSCs differentiation into astrocytes after several asymmetric divisions losing their stem cell capabilities (Encinas et al., 2011; Ziebell, Dehler, Martin‐Villalba, & Marciniak‐Czochra, 2018). One of the most intriguing aspects regarding the decline in adult neurogenesis is that even in an ever‐declining population, the proportion of activated NSCs is small (2%–5%), and therefore, the potential to maintain neurogenesis during aging exists by enhancing the recruitment of NSCs for activation. This can be experimentally achieved by increasing neuronal activity through electro‐convulsing shock (Segi‐Nishida, Warner‐Schmidt, & Duman, 2008); kainic acid (KA) injections (Huttmann et al., 2003; Sierra et al., 2015); or by reducing gamma‐aminobutyric acid (GABA) signaling to NSCs (Giachino et al., 2014; Song et al., 2012). We herein explore the properties of NSCs in aged mice to gain insight into these observations. We examined the morphology, the expression of markers, and the mitotic capacity of NSCs in 3‐, 12‐, and 18‐month‐old Nestin‐GFP mice, an extensively used transgenic line of mice in which NSCs can be readily visualized and identified.

## 2 RESULTS

### 2.1 The age‐induced loss of NSCs is accompanied by a change in morphological features

**Figure 1:**
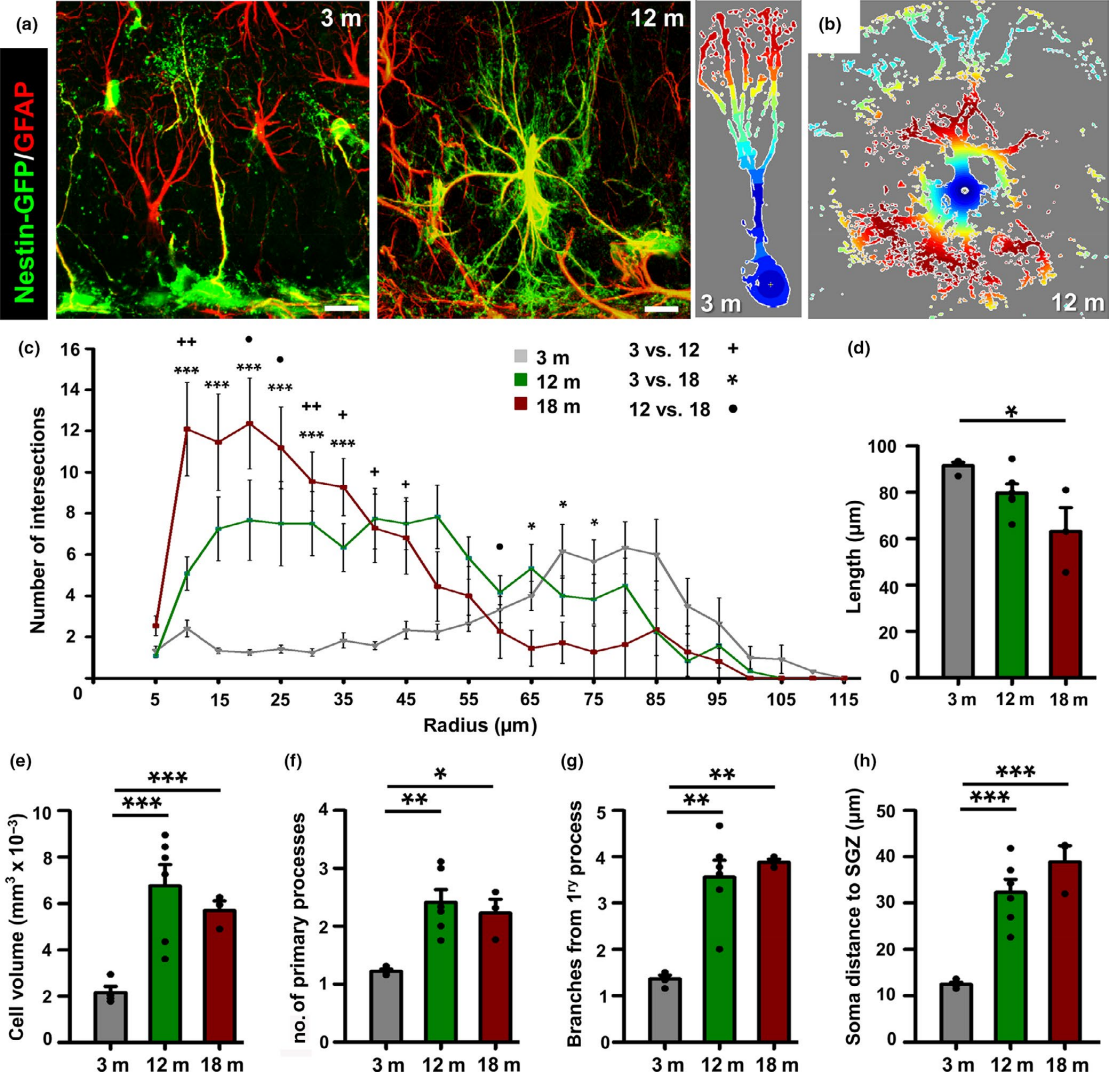
Neural stem cells (NSCs) drastically change their morphological properties with aging. (a) Confocal microscopy images showing the differences between NSCs in 3‐month‐old (3 m, left image) and 12‐month‐old (12 m, right image). Nestin‐GFP mice after staining for GFP (green) and GFAP (red). (b) Z‐stack projections of Nestin‐GFP+/GFAP+ cells prepared for 3D‐Sholl analysis. Color indicates distance from the center from closest (dark blue) to furthest (dark red). (c) Age‐associated differences in the complexity of NSCs (Nestin‐GFP+/GFAP+ cells). (d) Quantification of the length (center of soma to furthest tip of NSCs). (e) Quantification of the NSC volume. (f) Quantification of the number of NSC primary processes (those emerging from the soma). (g) Number of NSC secondary processes, defined as those branching from the primary process. (h) Quantification of the distance between the center of cell body and the SGZ. Scale bar is 10 μm in (a). *p < 0.05, **p < 0.01, ***p < 0.001 one‐way ANOVA after all pairwise multiple comparisons by Holm‐Sidak post hoc test (d‐h). 3 versus 12 m, ^+^ *p* < 0.05, ^++^ *p* < 0.01; 3 versus 18 m, *p < 0.05, **p < 0.01, ***p < 0.001; 12 versus 18 m, ^●^ *p* < 0.05, repeated measures ANOVA followed by Bonferroni post hoc test in c. Bars show mean ± *SEM*. Dots show individual data

We analyzed the total population of NSCs at different time points 3, 12, and 18 months old (m.o.) confirming the age‐associated decline of their number (Figure 1 and Supporting information Figure S1a,b). We used Nestin‐GFP transgenic mice, in which NSCs are readily visualized in the subgranular zone (SGZ) of the dentate gyrus (DG) (Mignone, Kukekov, Chiang, Steindler, & Enikolopov, 2004). NSCs are identified as radial glial‐like cells located at the SGZ, and with a long process crossing the granule cell layer (GCL) that ramifies into the molecular layer (ML, Figure 1a left image), these cells are positive for GFP (on the Nestin‐GFP transgenic line), nestin, GFAP, and brain lipid‐binding protein (BLBP) markers (Encinas, Vaahtokari, & Enikolopov, 2006; Kempermann et al., 2004; Seri et al., 2001). In aged mice (12 and 18 m.o.), the majority of Nestin‐GFP/GFAP cells remaining in the SGZ and the GCL presented a complex morphology with extensive branching and multipolar extensions similar to the reactive‐like morphology of reactive NSCs induced by hippocampal seizures (Sierra et al., 2015) (Figure 1a, right image) and in clear contrast to the typical morphology of type‐1 radial glia‐like NSCs (Figure 1a, left image).

To further investigate and quantify these differences in cell morphology, we analyzed the complexity of NSCs using 3D‐sholl analysis. Nestin‐GFP/GFAP NSCs within the SGZ and the GCL cells were randomly selected in order to measure morphological complexity by the quantification of the number of intersections between virtual spheres of increasing radius and the 3D reconstruction of the cell. We employed confocal‐imaging z‐stack reconstructions of NSCs (Figure 1b). This test was performed at the population level by analyzing random cells located in the SGZ + GCL, regardless of their morphology, to avoid any bias.

We observed a significant increase in the complexity in NSCs in aged mice (Figure 1c). Cells in 12‐ and 18‐month‐old mice presented a higher number of intersections within the 50 μm nearest to the nucleus than those NSCs from 3 m.o. mice, which in contrast presented more intersections in the distal portion of the cell, due to the profuse broccoli‐like arborization characteristic of radial NSCs.

We further analyzed morphological parameters such as length (measured as the distance from the center of the soma to the furthest cell tip), volume, or number of branches which can account for the increased morphological complexity associated with aging. NSCs in aged mice (18 m.o.) are significantly shorter than in younger (3 m.o.) mice (Figure 1d). The volume occupied by the NSCs in 12‐ and 18‐month‐old mice was threefold larger than in 3‐month‐old mice (Figure 1e). The number of primary processes, defined as the cytoplasmic prolongations that emerge directly from the soma, increased in aged mice being significantly higher in the two aged groups compared with the 3‐month‐old mice (Figure 1f). In contrast to the typical single radial process that characterizes NSCs in normal conditions of young mice, several primary processes emerged from the NSCs of aged mice (Figure 1a,b, and f). Frequently, basal ramifications emerging from the hilus‐oriented portion of the cell body were found in the NSCs of aged mice. In addition, the number of secondary branches that emerged from the primary process was almost threefold higher in NSCs than older mice (Figure 1g). Finally, we found also a difference in the location of the cell body of NSCs which in older mice was positioned into the GCL and further from the hilus (Figure 1h). These results show that with aging NSCs not only are reduced in number but in addition they switch drastically their morphology to a more complex and reactive‐like one.

### 2.2 Two types of morphologically NSCs are found in the aged DG

**Figure 2:**
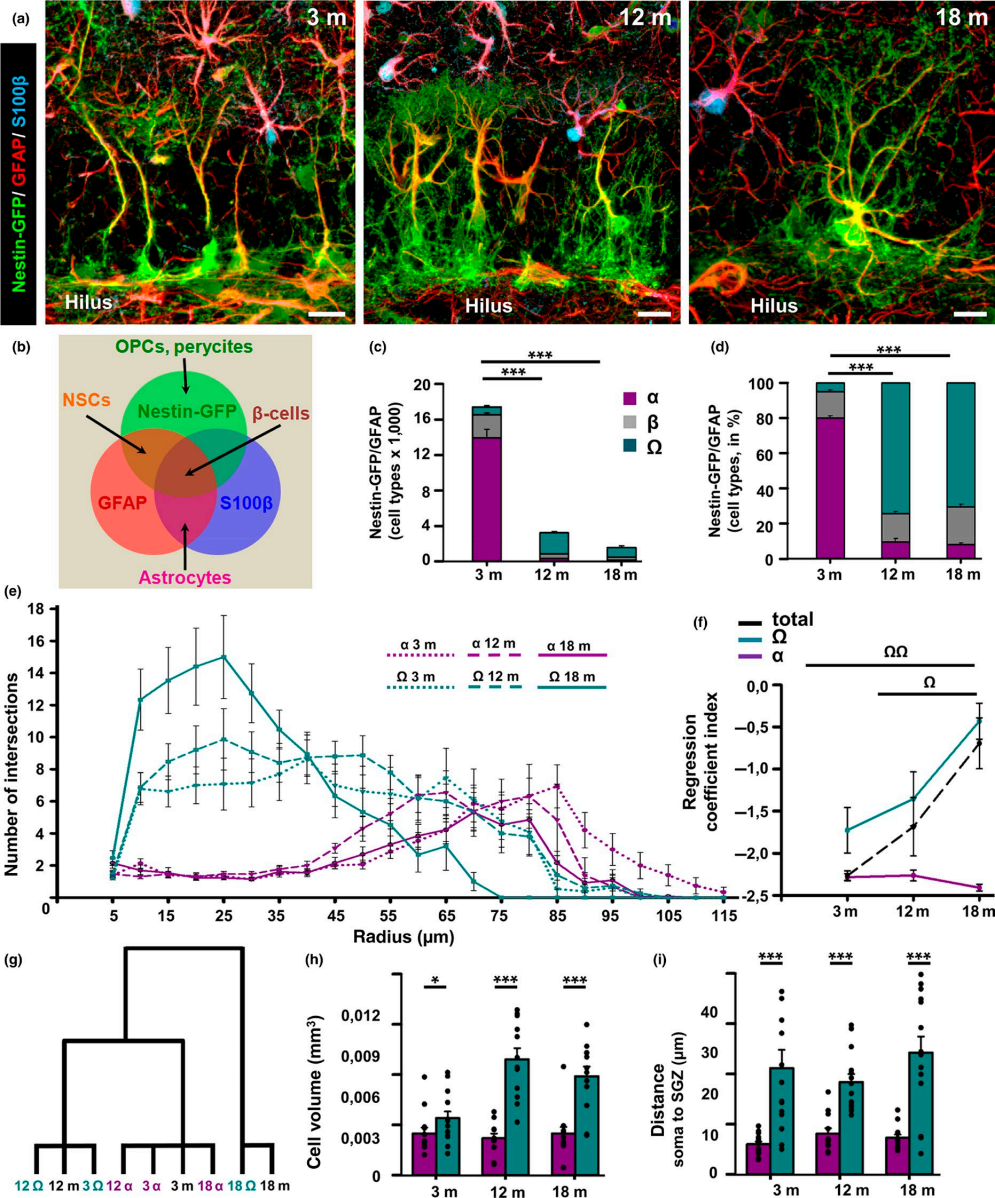
At least two populations of Nestin‐GFP+/GFAP+ cells are found in aged DG. (a) Confocal microscopy images (projection from z‐stacks) showing the increase in the complexity of neural stem cells (NSCs) over aging (3‐, 12‐, and 18‐month‐old mice). (b) Scheme showing the differential pattern of marker expression of cell types in the DG. (c) Quantification of the total number of the different NSC‐like populations (α, β, and Ω) in 3‐, 12‐, and 18‐month‐old mice. (d) Quantification of the different NSC‐like populations (α, β, and Ω) in percentage showing the significant decrease of α‐cells and the concomitant increase in the proportion of Ω‐cells. The β‐cell subpopulation remains relatively constant. (e) Quantification of the α‐ and Ω‐cell complexity by 3D‐Sholl analysis in each time point (see table for statistical analysis). (f) Quantification of the complexity index (*K*‐index) showing a significant increase of the complexity in Ω‐cells and in the total population of NSCs with age. (g) Ward's hierarchical clustering based on 3D‐Sholl analysis to independently classify α and Ω cells in each age point. (h) Quantification of the cell volume by cell type and age point. (i) Quantification of the distance of the cell body to the SGZ. Scale bar is 20 μm in (a). (e) *p < 0.05, **p < 0.01, ***p < 0.001 repeated measures ANOVA followed by Bonferroni post hoc test. (c, d and f) one‐way ANOVA after all pairwise multiple comparisons by Holm‐Sidak post hoc test. (h–i) *p < 0.05, **p < 0.01, ***p < 0.001 by Student's *t* test. Bars show mean ± *SEM*. Dots show individual data

We noticed that rather than a generalized change in complexity in all NSCs, at least two morphologically distinct subpopulations, and different location of the soma, of Nestin‐GFP/GFAP‐positive cells could be found. The first type corresponds to the typical radial astrocyte, type‐1, or radial NSCs whose morphological and functional properties have been extensively described (Encinas et al., 2011, 2006; Filippov et al., 2003; Mignone et al., 2004; Seri et al., 2001; Sierra et al., 2015). Their soma is located in the SGZ, and they extend a single radial process that crosses the GCL and arborizes profusely in the molecular layer (Figure 1a). For the sake of distinction and following previous nomenclature (Gebara et al., 2016), we will refer to these cells as “alpha” (α‐cells) from now onwards. The second phenotype corresponds to Nestin‐GFP/GFAP‐positive cells that are located predominantly in the GCL. These cells are more ramified, presenting several prolongations emerging from the soma toward the SGZ and the hilus. All their prolongations are thicker than in their α‐cell counterparts. We will refer to these cells as “omega” (Ω‐cells). Following these morphological criteria, these cells could be classified as “beta” (β‐cells), described recently (Gebara et al., 2016). However, in clear contrast to β‐cells, Ω‐cells do not express S100β (Figure 2a and Supporting information Figure S2a). In order to quantify the absolute cell numbers of the distinct populations of Nestin‐GFP/GFAP cells (Figure 2a) and their relative proportions over aging, we established objective and clear criteria to identify them. α‐cells present one single process emerging from the soma of at least 40 μm in length and that crosses the GCL bifurcating in the ML. Their cell body is located in the SGZ without any or very short (5 μm or less) basal processes. β‐cells: As previously characterized, these cells present a multibranched morphology; the cell body can be located either in the SGZ or in the GCL, and importantly, they express the S100β (Gebara et al., 2016). Ω‐cells present a multibranched morphology with several primary processes emerging from the soma. The cell body is located in the GCL, and they bear basal processes that extend from the soma toward the SGZ or hilar region. They do not express S100β. To facilitate the understanding of the distinction among the cell of interest and other that form part of the hippocampal neurogenic niche, a scheme summarizing the differences in marker expression is shown in Figure 2b.

The population of α‐cells declined sharply, from several thousands to a few hundred (12 m.o.) or dozens (18 m.o.) of cells with aging (Figure 2c). β‐cells followed a similar trend, but their absolute numbers were much lower already in the young mice (Figure 2c). In contrast, Ω‐cells increased significantly the size of its population from 3 to 12 months of age and then declined in the 18‐month‐old group although remaining above the 3‐month‐old mice (Figure 2c). Besides the lack of expression of S100‐ß and the expression of Nestin‐GFP and GFAP, α‐ and Ω‐cells shared the expression of other radial glia/NSC markers such as sex determining region Y‐box 2 (Sox2), lysophosphatidic acid receptor 1 (LPA1), and brain lipid‐binding protein (BLBP) (Supporting information Figure S2e‐j). Although the expression of Sox2 and BLBP suggests a close lineage relationship between α‐ and Ω‐cells, no claims can be made regarding their “stemness” as Sox2 and BLBP are also present in hippocampal astrocytes. In turn, LPA1 has been recently described as a very specific marker of NSCs in the adult hippocampus (Walker et al., 2016).

When considering the proportion of each cell type to the total population of Nestin‐GFP/GFAP‐positive cells, a fundamental change was observed. While in 3‐month‐old mice α‐cells clearly comprised the majority of the NSCs population, it converted into the smallest subpopulation in 12‐ and 18‐month‐old animals (Figure 2d). Conversely, Ω‐cells represented the smallest relative proportion in the 3‐month‐old mice, but were the largest subpopulation in the animals of 12 and 18 months of age (Figure 2d). β‐cells represented a more stable relative population over time (Figure 2d). We focused our study on α and Ω‐cells, as β‐cells have already been well‐characterized, because they lack cell division and their expression of S‐100β suggests that they are a transitional step toward astrocytic differentiation (Gebara et al., 2016). We moved on to analyze the complexity of α and Ω‐cells separately by 3D‐Sholl analysis in each age point. We found that the complexity of α‐cells did not change regardless of the age of the animals, whereas the complexity of the Ω‐cell population significantly increased over time (Figure 2e). A readier visualization of this change can be obtained by plotting Sholl regression coefficient (*k*‐index), which is a measure of the rate of decay of intersections with their distance to the cell body center. Higher *k* values reflect larger changes, which is translated in higher complexity (Ferreira et al., 2014) at the different age points (Figure 2f). The complexity of Ω‐cells was higher than that of α‐cells at 3 months of age, and it further increased at 12 months of age, being even higher at the 18‐month‐old age point, whereas it did not change for α‐cells. When considering the total population of Nestin‐GFP/GFAP‐positive cells (and S100β‐negative), the complexity *k*‐index change parallels that of Ω‐cells (Figure 2f). This can be attributed to the fact that with age the population of α‐cells gets depleted and Ω‐cells increase their number and their proportion. We performed Ward's hierarchical clustering (Ferreira et al., 2014) to independently classify α‐cells and Ω‐cells, and compare them with the whole population of NSCs (Figure 2g). The analysis yielded three groups. α‐cells at 3, 12, and 18 months of age clustered together with the total NSC population at 3 months, reflecting the fact that α‐cells account for most of the NSCs in younger animals. Ω‐cells at 3 and 12 months clustered with the total NSCs population at 12 months, reflecting how their morphology is clearly different form that of α‐cells and actually account for most of the NSC population at 12 months of age. Finally, Ω‐cells at 18 months clustered together with the total population at 18 months, reflecting not only that Ω‐cells are the most abundant cell type at this age point, but also that their complexity changes with aging (Figure 2g).

We then measured several morphological parameters that could account for the observed changes in complexity. Ω‐cells bear more processes emerging directly from the soma (primary processes) than α‐cells (Supporting information Figure S2b), and these primary processes bifurcate with higher frequency (Supporting information Figure S2c). The length of Ω‐cells is also significantly shorter than that of α‐cells (Supporting information Figure S2d). In addition, the cell volume is larger for Ω‐cells (Figure 2h) and the distance of the cell soma to the SGZ is longer (Figure 2i). These results are the same for all the age points analyzed.

These results show that there are two populations of Nestin‐GFP/GFAP‐positive, S100β‐negative cells in the aging DG. While the extensively described α‐cells decline sharply in number, the morphologically distinct population of Ω‐cells increases with aging and comprises by large the most abundant population in aged mice. In addition, the Ω‐cell population changes its morphology overtime. These findings suggest that α‐cells transform into Ω‐cells with aging, and that the development of a reactive‐like complex morphology is a progressive time‐dependent process.

To assure that the categorization between α‐ and Ω‐cells can be performed in an unbiased and reproducible manner in our own laboratory and elsewhere, we developed a freely available pack of macros for ImageJ named “CellShaper” which facilitates the selection of the cells, automatically performs Sholl analysis, and classifies selected cells in two categories (omega and alpha) in an automatized and unsupervised manner (Supporting information Figure S3c and available for download here: https://www.achucarro.org/downloads). A match of 94.75% between the manual and the automatized classification was achieved.

### 2.3 Ω‐cells are more quiescent than α‐cells

**Figure 3:**
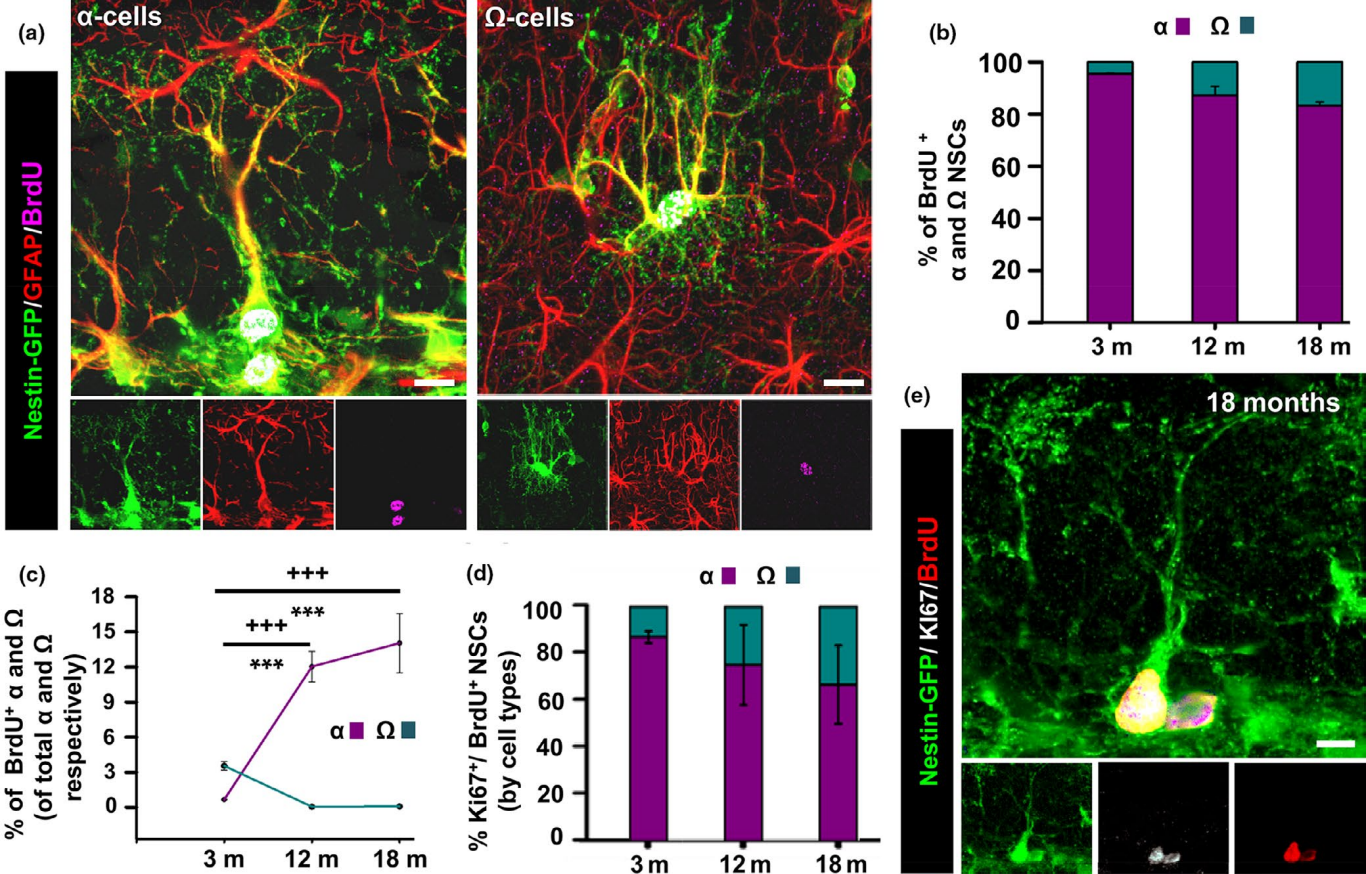
Ω‐cells divide with lower probability. (a) Representative confocal microscopy images of dividing Nestin‐GFP+/GFAP+ α‐NSC after staining for GFP, BrdU, and GFAP at 3 and 18 months old. (b) Quantification of the cell division (incorporation of BrdU) in the different subpopulations of neural stem cells (NSCs). (c) Quantification of the proportion of dividing α and Ω‐cells, among the total α and Ω‐cell population, at each age point. (d) Quantification of the re‐entry into the cell cycle of NSCs (Ki67‐ and BrdU‐positive NSCs). (e) Confocal microscopy image (projection from z‐stacks) staining for GFP, BrdU, and Ki67. Scale bar is 10 μm in (a and g). ***p < 0.001 one‐way ANOVA after all pairwise multiple comparisons by Holm‐Sidak post hoc test. Bars show mean ± *SEM*. Dots show individual data

We next sought to investigate whether the two morphologically distinctive populations had also different functional properties. We administrated the thymidine analogue 5‐bromo‐2‐deoxyuridine (BrdU, four injections 2 hr apart) to mice of the three different age points considered and sacrificed them 24 hr later to assess cell proliferation (Figure 3a). As expected, the overall number of BrdU‐labeled cells is drastically reduced with aging in the DG (Supporting information Figure S3a,b). We found that the capability to incorporate BrdU is almost restricted to type α‐cells which therefore account for most of the dividing NSCs at any analyzed age point. However, Ω‐cells retained some mitotic potential (Figure 3b) and incorporated BrdU although with very low frequency. At all the different age points, α‐cells accounted for the vast majority of Nestin‐GFP/GFAP cells that incorporated BrdU, while the fraction of BrdU+ Ω‐cells was much lower (Figure 3b). In both cases, the majority of the cells comprising each population remained quiescent, but interestingly, α‐cells were able to increase their activation, in percentage over the total α‐cell population, over time as shown by the significantly higher proportion of BrdU‐labeled cells among the α‐cells population in aged mice (Figure 3c). In contrast, the proportion of activated (BrdU‐incorporating) Ω‐cells significantly decreased in aged mice (Figure 3c). To further analyze α and Ω‐cell mitotic activity, we next analyzed the capacity of NSCs to re‐enter the cell cycle by measuring the proportion of BrdU‐positive NSCs that expressed the mitotic marker Ki67 among the total number of BrdU‐positive NSCs of each type. Similarly to the results obtained for cell activation, we found that Ω‐cells re‐entered the cell cycle with significantly lower probability than α‐cells, regardless of the age point (Figure 3d,e). These results combined strongly suggest that Ω‐cells have markedly reduced mitotic potential compared to α‐cells and that not only they are activated with lower rate but also the probability of undergoing another round of cell division within the next 24 hr is decremented (Figure 3d,e).

While the remaining α‐cells in the aged DG increase their activation rate, Ω‐cells become even more quiescent with aging. The results support the notion that rather than two distinct populations generated independently, Ω‐cells are derived from α‐cells, and together with a increase in reactive‐like morphology they get activated with lower probability.

### 2.4 Ω‐cells remain mostly quiescent even in pro‐activation conditions

**Figure 4:**
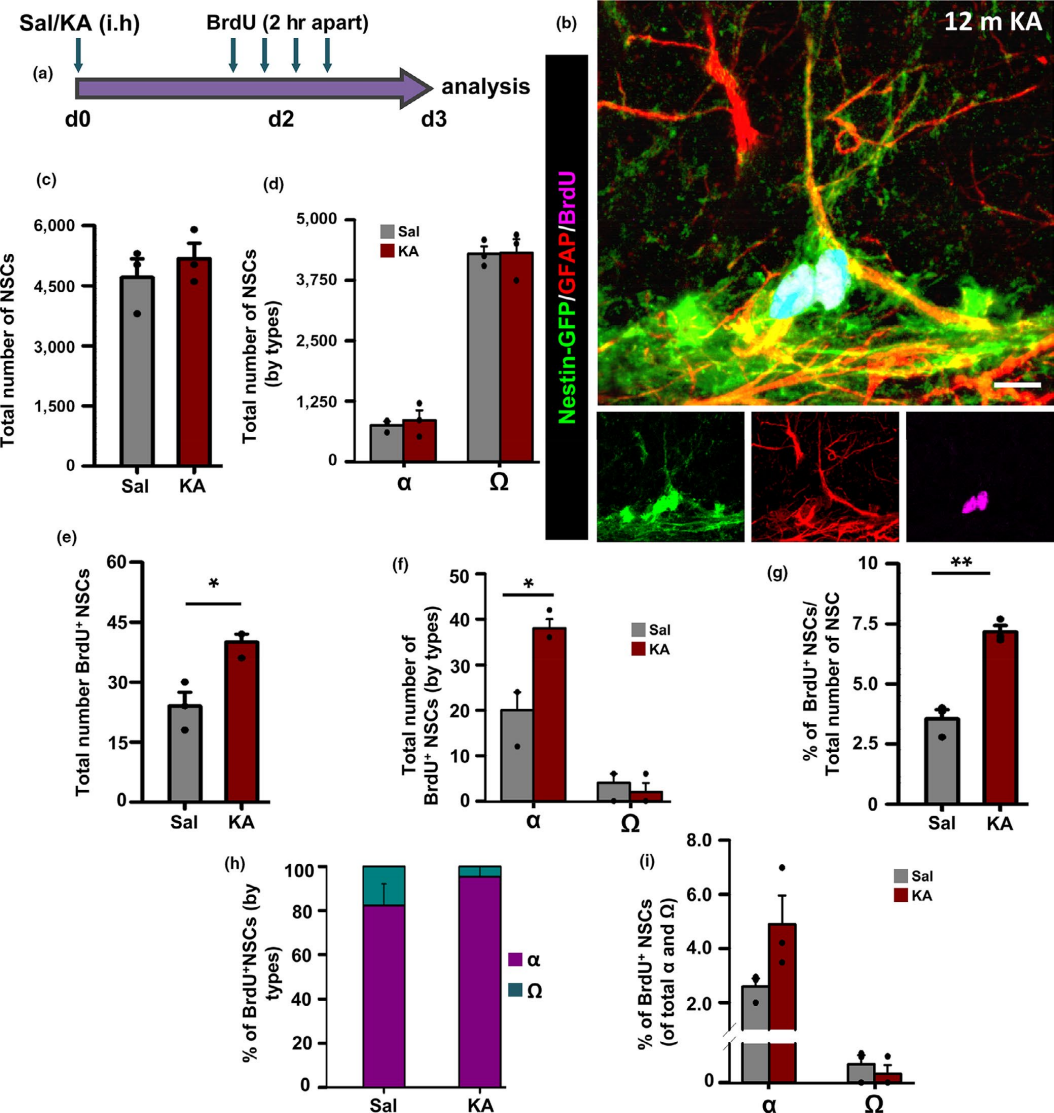
Ω‐cells are more quiescent even in pro‐activation conditions. (a) Experimental paradigm of Sal/KA and BrdU administration to 12‐month‐old animals. (b) Confocal microscopy projections of z‐stacks showing a dividing α‐NSC after immunostaining for GFP, GFAP, and BrdU. (c) Quantification of the total number of neural stem cells (NSCs). (d) Quantification of the total number of NSCs by subpopulations. (e) Quantification of total dividing NSCs. (f) Quantification of the total number of dividing NSCs by subtypes. (g) Quantification of the proportion of total dividing NSCs. (h) Quantification of the proportion of the dividing NSCs by subpopulations. (i) Quantification of the proliferative capacity of α and Ω‐NSCs of the total α and Ω‐NSC populations. Scale bar is 10 μm in B. *p < 0.05, **p < 0.01, ***p < 0.001, *p < 0.05, and ***p < 0.001 by Student's *t* test. Bars show mean ± *SEM*. Dots show individual data

We wondered whether Ω‐cells retained at least plasticity to respond to a pro‐activation stimulus and adapt their rate of mitosis to neuronal activity as α‐cells are able to do (Sierra et al., 2015). We subjected mice of 12 months of age to intrahippocampal injection of the glutamatergic agonist KA in a dose that triggers neuronal hyperactivation (without inducing seizures or cell death) and a subsequent increase in cell proliferation (Supporting information Figure S3c,d) and in the number of NSCs that enter the cell cycle. To be even surer that this procedure did not trigger alterations in the niche such as the induction of reactive NSCs, we analyzed the contralateral hemisphere. KA was injected in the right hippocampus of 12‐month‐old mice, and after 2 days, the animals received BrdU (four injections 2 hr apart). Twenty‐four hours after the last injection, the animals were sacrificed and the left hemisphere was analyzed (Figure 4a,b). We chose the 12‐month age point to have a sufficient number of Ω‐ and α‐cells to analyze and to avoid anesthesia and surgery‐related problems in the most aged mice. The total number of NSCs was not changed between the control injected with saline and the experimental (12‐month‐old KA) group (Figure 4c). Further, analyzing separately the total number by subtypes, in both conditions, the same number of α and Ω‐cells was found (Figure 4d). We confirmed that intrahippocampal KA significantly activated more NSCs (Figure 4e), which was translated into a higher proportion of BrdU‐incorporating NSCs (Figure 4g). While the number of activated α‐cells increased significantly in the KA‐injected mice, the number of Ω‐cells incorporating BrdU remained unchanged (Figure 4f). α‐cells accounted for most of the dividing Nestin‐GFP/GFAP NSCs in controls, and this proportion was further elevated in KA‐injected mice (Figure 4h). This change was also observed when considering the proportion of dividing α‐ and dividing Ω‐cells among the total population of α‐ and Ω‐cells, respectively (Figure 4i). These results confirm that Ω‐cells are more quiescent than α‐cells and are unable to increase their rate of mitosis even in pro‐activation conditions such as neuronal hyperactivation and that thus they are a different morphologically and functionally population.

### 2.5 Chronic inflammation induces the conversion of α‐cells into Ω‐cells

**Figure 5:**
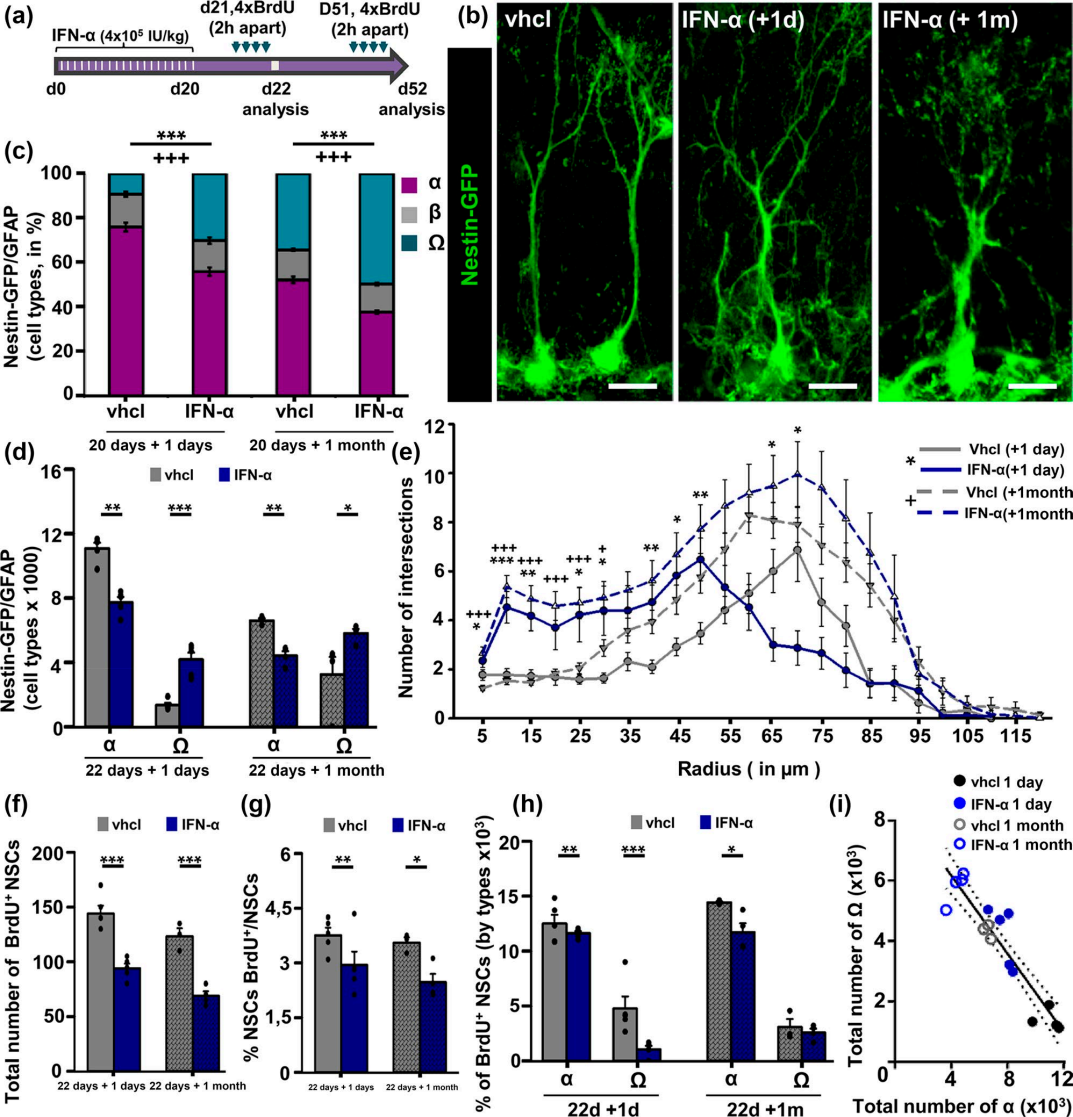
Chronic inflammation converts α‐cells into Ω. (a) Experimental paradigm of Sal/IFN‐α and BrdU administration. (b) Confocal microscopy projections of neural stem cells (NSCs) after immunostaining for GFAP and GFP. (c) Quantification of the different subtypes of NSCs. (d) Quantification of the changes in NSC complexity. (e) Quantification of the total number of α and Ω cells. (f) Quantification of the dividing NSCs. (g) Quantification of the proportion of dividing NSCs. (h) Quantification of the dividing NSCs by subtypes. Straight line fitting of the data showing that Ω cells significantly and negatively correlated with the number of α cells. Scale bar is 10 μm in (b). (e) *p < 0.05, **p < 0.01, ***p < 0.001 for comparisons at 1 day after treatment; ^+^ *p* < 0.05, ^++^ *p* < 0.01, ^+++^ *p* < 0.001 for comparisons at 1 month after treatment. Repeated measures ANOVA followed by Bonferroni post hoc test. (c, d, and f–i) *p < 0.05, **p < 0.01, ***p < 0.001, *p < 0.05, and ***p < 0.001 by Student's *t* test. Bars show mean ± *SEM*. Dots show individual data

Next, we wanted to confirm whether as suggested by the previous results those α‐cells that remains in the DG transform into Ω‐cells overtime, rather than α‐cells and Ω‐cells comprising two different populations established early on. We hypothesized that chronic neuroinflammation, a hallmark of brain aging (Lynch, 2010), might be playing a role in the accumulation of Ω‐cells as neuroinflammation is antineurogenic and reduces the activation of NSCs (Ekdahl, Claasen, Bonde, Kokaia, & Lindvall, 2003; Sierra et al., 2015). To test this hypothesis, we injected 3‐month‐old Nestin‐GFP mice intraperitoneally with a low dose of interferon‐α (IFN‐α, 4 × 10^5^ IU/kg) during 20 days in order to mimic the chronic inflammation of the aged brain (Zheng et al., 2014; Figure 5a). 24 hr after the last injection of IFN‐α, the mice received 4 injections of BrdU (2 hr apart) and were analyzed 24 hr after the last one (Figure 5a,b). We chose the 3‐month‐old time point because at this age the α‐cells population is largely predominant (Figure 2). The total number of NSCs was not changed compared to the control (Supporting information Figure S4a). Interestingly, the proportion of α‐cells was significantly reduced in animals injected with IFN‐α, while the proportion of Ω‐cells was significantly increased (Figure 5c). The proportion of β‐cells remained unchanged.

We then analyzed overall Nestin‐GFP/GFAP cell complexity by 3D‐Sholl analysis in a random manner, that is, without making any selection by cell types. Morphological complexity was found to be significantly higher in IFN‐α‐injected mice than in the control (saline‐injected) mice (Figure 5b,d). Furthermore, the number of primary processes and the number of branches emerging from the primary processes were significantly increased in IFN‐treated mice (Supporting information Figure S4b,c). NSCs from the IFN‐α mice had also increased cell volume (Supporting information Figure S4d); and their soma was localized into the GCL further from the SGZ (Supporting information Figure S4e) repeating the same pattern observed in aged mice (Figure 2). We next focused on α‐ and Ω‐cells, using the criteria defined above, and found that the total number of α‐cells was significantly diminished in the IFN‐α mice, whereas the number of Ω‐cells was significantly increased (Figure 5e). Furthermore, in the IFN‐α‐injected mice the number of newly generated Ω‐cells was very similar to the number of lost α‐cells. We analyzed whether IFN‐α promoted functional changes by analyzing BrdU incorporation as a measure of mitotic activity. IFN‐α reduced the overall activation of NSCs in absolute numbers (Figure 5f) and in relative proportion (Figure 5g). Interestingly, when analyzed by subtypes we found that the proportion of activated α‐cells (among the total of BrdU cells) did not change but that the proportion of dividing Ω‐cells was significantly decremented, thus accounting for the overall reduction in Nestin‐GFP/GFAP cell activation as Ω‐cells are more abundant IFN‐α mice (Figure 5h). Therefore, IFN‐α is not only triggering the morphological and anatomical changes in α‐cells but is also inducing quiescence, a main characteristic of Ω‐cells (Figure 3). These results support our hypothesis that Ω‐cells are derived from those α‐cells that do not get depleted over time and remain in the DG. In addition, they suggest that chronic inflammation could be playing a role in the conversion of α‐cells into Ω‐cells and therefore in the reduction of neurogenesis associated with aging. We were also interested in testing how reversible was the induction of Ω‐cells by IFN‐α. Three‐month‐old Nestin‐GFP mice were injected with either vehicle or IFN‐α for 20 days and then allowed 30 days of recovery before BrdU administration (day 51) and sacrifice (day 52) (Figure 5a,b). We proceed to check the same parameters as for the no‐recovery mice (number and proportion of α‐ and Ω‐cells, Figure 5c,d; morphological complexity Figure 5e; correlation between α‐ and Ω‐cells, Figure 5f and mitotic activity, Figure 5g,h and i) and found that all the effects induced by IFN‐α were maintained in spite of the recovery time. This result suggests the conversion of α‐cells into Ω‐cells is an irreversible process. Importantly, considering data from both saline and IFN‐α injected mice, both with and without recovery, the number of Ω‐cells significantly and negatively correlated with the number of α‐cells (Pearson correlation coefficient *r* = 0.93). In addition, we performed a regression analysis and found that data best fitted to a linear regression model (*R* ^2^ = 0.87, *p* ˂ 0.0001; Figure 5i, and Supporting information Figure S4f). This result suggests that Ω‐cells are able to directly derived from α‐cells.

### 2.6 Anti‐inflammatory treatment with minocycline does not prevent the conversion of α‐ into Ω‐cells

**Figure 6:**
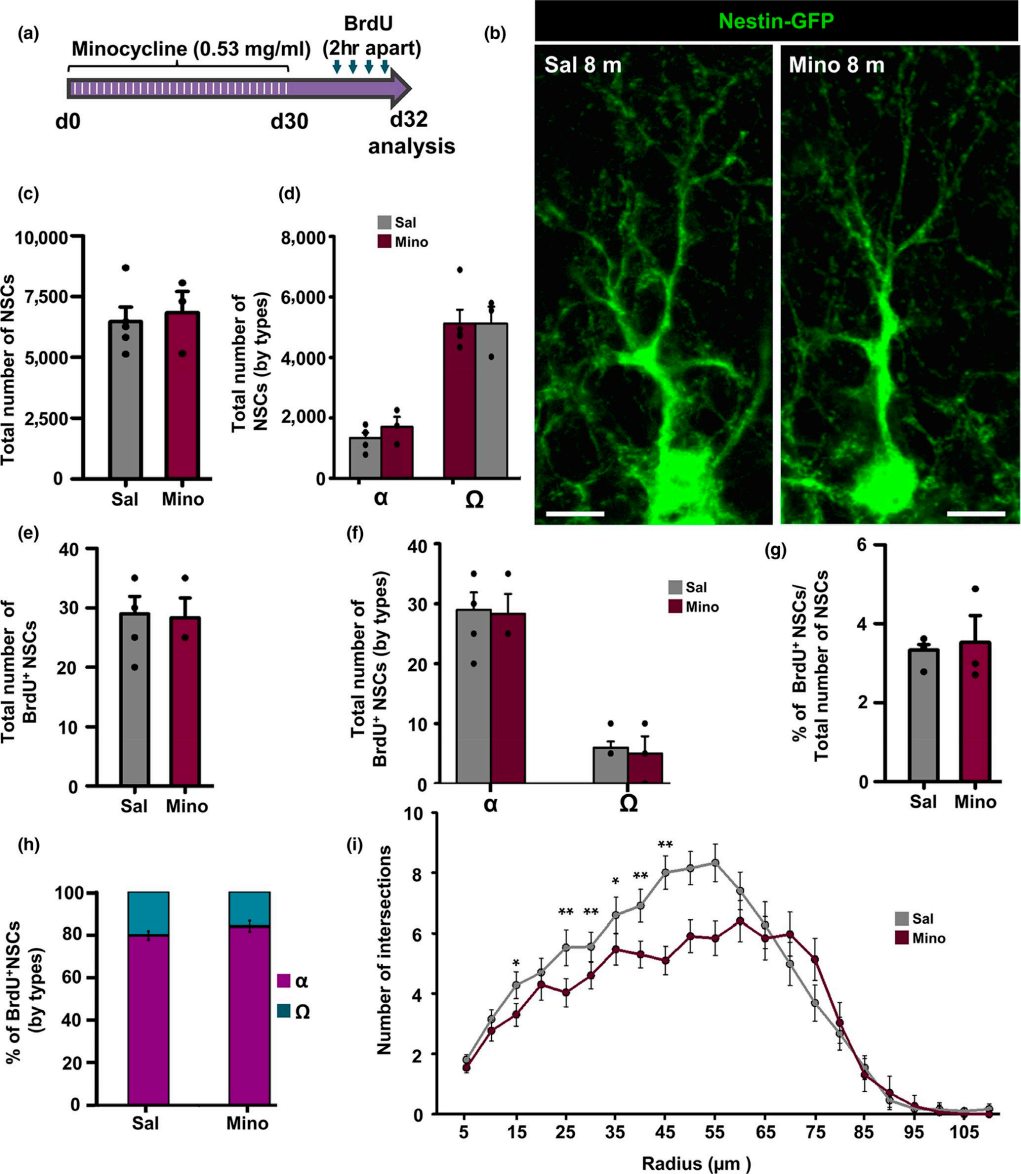
Chronic anti‐inflammatory treatment does not revert the conversion of α‐ into Ω‐cells. (a) Experimental paradigm of minocycline (30 days) and BrdU administration (one day before sacrifice) to 8‐month‐old animals. (b) Confocal microscopy projections of Nestin‐GFP‐labeled neural stem cells (NSCs). (c) Quantification of the total number of NSCs. (d) Quantification of the total number of NSCs by subpopulations. (e) Quantification of total dividing NSCs. (f) Quantification of the total number of dividing NSCs by subtypes. (g) Quantification of the proportion of total dividing NSCs. (h) Quantification of the proportion of the dividing NSCs by subpopulations. (d) Quantification of the changes in NSC morphological complexity. This is the only parameter in which administration of minocycline has an effect. Scale bar is 10 μm in b. *p < 0.05, **p < 0.01. Repeated measures ANOVA followed by Bonferroni post hoc test in (i). Student's *t* test in (e–h). Bars show mean ± *SEM*. Dots show individual data. *n* = 5 in control and *n* = 4 in minocycline

We wondered next whether an anti‐inflammatory treatment could prevent or ameliorate the generation of Ω‐cells. We administered minocycline a semi‐synthetic tetracycline with anti‐inflammatory effects in the brain (Kohman, Bhattacharya, Kilby, Bucko, & Rhodes, 2013) in drinking water for 30 days (Figure 6a,b) to 8‐month‐old mice. We chose this age so that sufficient numbers of α‐ and Ω‐cells were present and changes in either direction could be observed. We did not find any change due to the administration of minocycling in the number of NSCs, total (Figure 6c) of by subtypes (Figure 6d). The total number of dividing NSCs (Figure 6e), or α‐ and Ω‐cells (Figure 6f) or in their relative proportions (Figure 6g,h) were not altered by minocycline. Only morphological complexity was decreased over the vehicle‐treated mice (Figure 6b,i). These results suggest that indeed even though inflammation might be playing a role in the potential conversion of α‐cells into Ω‐cells but might not be the only factor associated with aging involved.

## 3 DISCUSSION

The age‐related decrease of neurogenesis is well described in the DG of experimental animals from rodents to primates (Kuhn et al., 1996; Leuner, Kozorovitskiy, Gross, & Gould, 2007; Ngwenya, Heyworth, Shwe, Moore, & Rosene, 2015; Walter et al., 2011). The depletion of NSCs population might be the main force driving the decrease of neurogenesis with age (Encinas et al., 2011; Walter et al., 2011). That activation of NSCs could translate into their depletion was suggested by the in vivo blockade of inhibiting bone morphogenetic protein (BMP) signaling (Mira et al., 2010). It was later shown that depletion of the NSC population was directly linked to their recruitment to enter into the cell cycle with the underlying mechanism being astrocytic differentiation of NSCs after several rounds of asymmetric division to generate neuronal fate‐committed progenitors (Encinas et al., 2011). Preventing activation of NSCs by the inducible knock down of Ascl‐1 (Mash‐1) in NSCs indeed prevented the decline of the NSCs population (Andersen et al., 2014). Recent data obtained using mathematical modeling show that activation‐coupled astrocytic differentiation after activation alone might not account for all the depletion of the NSC pool and that other mechanisms, might be apoptosis, could play an important role (Ziebell et al., 2018). Although NSCs can undergo symmetric division to generate more copies of themselves, this process is not frequent enough to counterbalance in normal conditions the age‐associated decline of NSCs (Bonaguidi et al., 2011). In vivo two‐photon imaging of neurogenesis in the hippocampus has confirmed most of these observations with unprecedented resolution and has yielded new findings: Hippocampal NSCs might comprise several subpopulations with different behaviors regarding asymmetric versus symmetric division, neurogenic versus astrocytic differentiation, and difference in the period of quiescence between mitosis (Pilz et al., 2018). Noteworthy, depletion of NSCs alone cannot explain the loss of generation of neuronal progenitors that eventually give rise to newborn neurons. As the number of activated NSCs is very small compared to the total size of the population, the number of activated NSCs (and subsequently the number of neuronal progenitors) could remain constant for longer periods of time even if the total size of the NSC population declines. The amount of neurogenesis in the human hippocampus is currently subject of intense debate with data supporting the generation of newborn neurons through aging (Eriksson et al., 1998; Spalding et al., 2013) in a more or less steeper decline (Boldrini et al., 2018; Moreno‐Jiménez et al., 2019; Spalding et al., 2013) or with a dramatic decline similar to rodents (Knoth et al., 2010). Reduction of neurogenesis to undetectable levels in the human hippocampus after infancy has also been reported (Cipriani et al., 2018; Dennis, Suh, Rodriguez, Kril, & Sutherland, 2016; Sorrells et al., 2018). In any case, the key to explain these results altogether resides in how sharp is the loss of neurogenesis. As NSCs represent the first step of the neurogenic cascade and in net terms can be considered a nonrenewable source of neurons, understanding how their properties change overtime is essential to elucidate how hippocampal neurogenesis declines with age.

In this paper, we described that not only the total number of NSCs decreases with age but also that the remaining NSCs change their morphology into a more complex one. Using a combination immunohistochemistry, morphological parameters, and the tridimensional analysis of NSCs, we found a new phenotype of NSC coexisting with the described type α‐ and β‐cells (Gebara et al., 2016). This new subtype, that we called herein Ω‐cells for the sake of clarity, presents a significant increase in the complexity characterized by a multibranched morphology with several primary processes, a decrease of the length and the cell body located in the SGZ + GCL. Ω‐cells divide with much lower probability as measured by the incorporation of BrdU. The lack of S100β expression in Ω‐cells differentiates them from the astrocyte‐like β‐cells, and the expression of nestin (Nestin‐GFP) and LPA1 supports their close relationship with α‐NSCs. Furthermore, even in pro‐activation conditions as KA injections (Sierra et al., 2015) Ω‐cells are the less proliferative subpopulation. We cannot exclude the possibility that the response to other proneurogenic stimuli could be different.

Our description of a new subtype of NSCs shed light on the behavior of NSCs in aged DG, because as we described, whereas the α‐cells decline sharply in numbers from several thousands to a few hundred cells, in agreement with previous reports (Encinas et al., 2011; Walter et al., 2011), but in contrast Ω‐cells increase their numbers and comprise, by large, the most abundant population in aged mice.

This morphological change is directly related to aging, as it was showed in the populational profile changes. While in young animals the proportion of α‐cells comprised the majority of the NSCs population, in old animals α‐cells represented the smallest relative proportion, being Ω‐cells the main subtype in older mice. We hypothesize that rather than two different populations generated independently, a subpopulation of α‐cells senesces into Ω‐cells. Our data from IFN‐α‐treated mice support this idea. Mice injected with IFN‐α showed a very similar total number of NSCs compared to saline‐injected mice. IFN‐α induced, however, a reduction in the number of α‐cells and a proportional increase in Ω‐cells, thus suggesting a conversion from α‐ to Ω‐cells. Indeed, our analysis shows a high level of correlation between the α‐cells that disappear and the Ω‐cells that are generated. Therefore, α‐cells could transform into Ω‐cells with aging, developing a more complex morphology progressively in a time‐dependent process, diminishing their proliferative capacity at the same time. A 1‐month‐long period after IFN‐α did not cause recovery of α‐cell numbers, activation or morphology suggesting the irreversibility of the conversion of α‐ into Ω‐cells. These results, however, do not prove that a direct transformation of α‐cells into Ω‐cells takes place in natural aging. Recently, mathematical models described that the age‐related depletion of the NSCs might be explained because stem cells spend longer time in quiescence during aging (Ziebell et al., 2018). This model also predicts that the probability of exiting the quiescent stage through activation versus depletion increases during aging, which fits with our results. The molecular changes that occur in NSCs population during aging remain to be elucidated, but this work provides a framework for future research. It is known that in aged mice the genes associated with inflammation and oxidative stress are upregulated compared to young mice (Lynch, 2010) and one of the main effects of the neuroinflammation is the reduction of neurogenesis (Ekdahl et al., 2003; Gebara et al., 2016; Sierra et al., 2015). We demonstrated that the chronic administration of IFN‐α mimics the effect of aging as IFN‐α increases the quiescence of NSCs and induce or at least contributes significantly to the conversion of α cells into Ω‐cells. Although neuroinflammation is a multifactorial phenomenon and responses to other cytokines could be different, these findings give us reasons to hypothesize that chronic inflammation could mediate the acquisition of Ω phenotype and increase the quiescence in NSCs. According to several studies, the lengthy treatment with IFN‐α affects directly cell proliferation in the SGZ, thus reducing hippocampal neurogenesis (Kaneko et al., 2006). In fact, the direct administration of this molecule diminishes specifically the neurogenic function of NSCs in the DG (Zheng et al., 2014), confirming our data. We tested the effect of the administration for 30 days of anti‐inflammatory agent minocycline and found no effect on cell number or cell activation. Morphology however was partially recovered. Inflammation is a complex multifactorial process, and further experiments will be needed to explore its interrelationship with the aging of NSCs.

Our results shed light about how aging affects DG neurogenesis and in particular how NSCs may be affected by aged‐related inflammation (inflammaging) because it is not only the “deforestation” of NSCs but also the correlated emergence of a senescent phenotype associated with chronic inflammation that explains the decrease of neurogenesis with aging.

## 4 EXPERIMENTAL PROCEDURES

### 4.1 Animals

All the experiments were performed employing Nestin‐GFP transgenic mice (Mignone et al., 2004).

All procedures were approved by the University of the Basque Country (EHU/UPV) and the Comunidad Foral de Bizkaia Ethics Committees (CEEA: M20/2015/236). All procedures followed the European directive 2010/63/UE and NIH guidelines. The mice were 3, 12, and 18 months old at the time of analysis and BrdU administration. The administration of IFN‐α or saline started when the mice were 2 months old. The injection of KA or saline was performed in 12‐month‐old animals.

BrdU and IFN‐α administration, and intrahippocampal injection of KA were applied following standard protocols for intraperitoneal injection and stereotaxic surgery, respectively (Sierra et al., 2015).

### 4.2 Immunohistochemistry

Experiments were performed as described before following standard procedures optimized for the use in Nestin‐GFP transgenic mice (Encinas et al., 2011).

### 4.3 Image capture

All fluorescence immunostaining images were collected employing a Leica SP8 (Leica, Wetzlar, Germany) laser scanning microscopes and their corresponding manufacturer's software following protocols optimized for stereotaxic quantification and quantitative image analysis (Encinas et al., 2011; Sierra et al., 2015). Images were captured in systemized random manner (see Extended Experimental Procedures).

### 4.4 Cell quantification and Sholl analysis

Quantitative analysis of cell populations in Nestin‐GFP mice was performed by design‐based (assumption free, unbiased) stereology using a modified optical fractionator sampling scheme as previously described (Encinas et al., 2011,2006). For the morphological analysis of NSCs, we obtained 3D reconstructions from confocal stack images (1,024 pixels of resolution). Single NSCs were analyzed using the 3D‐Sholl analysis plugin (http://fiji.sc/Sholl_Analysis) as described in Ferreira et al. (2014). A macro has been developed for the unsupervised automatized classification of NSCs (see details in Extended Experimental Procedures section in Supporting information). The macro is available for download here: https://www.achucarro.org/downloads.

### 4.5 Statistical analysis

sigmaplot (San Jose, CA, USA) was used for statistical analysis. When interaction between factors (age × treatment) was found, a 1‐way ANOVA test of all groups was performed instead. For analysis of pairs of groups, a Student's *t* test was performed. Hierarchical clustering was performed using Ward's method and squared Euclidean distances as linkage metric. For the Sholl analysis, two‐way repeated measures ANOVA followed by Bonferroni post hoc test was performed. The fitting of linear and nonlinear regression models to data was compared in graphpad prism 5 (GraphPad Software, Incl, San Diego, CA).

## CONFLICT OF INTEREST

None declared.

## AUTHORS CONTRIBUTIONS

Experiments were planned and designed by SMS and JME. Experimental data were generated and collected by SMS, JV, TMG and JME. Data analysis and interpretation involved SMS, JV, TMG and JME. Article draft was written by SMS, JV and JME. Critical revisions of the manuscript were performed SMS, JV and JME. Approval of the final version to be submitted by SMS, JV, TMG and JME.

## References

[acel12958-bib-0001] Andersen, J. , Urbán, N. , Achimastou, A. , Ito, A. , Simic, M. , Ullom, K. , … Guillemot, F. (2014). A transcriptional mechanism integrating inputs from extracellular signals to activate hippocampal stem cells. Neuron, 83(5), 1085–1097. 10.1016/j.neuron.2014.08.004 25189209 PMC4157576

[acel12958-bib-0002] Ben Abdallah, N.‐M.‐B. , Slomianka, L. , Vyssotski, A. L. , & Lipp, H.‐P. (2010). Early age‐related changes in adult hippocampal neurogenesis in C57 mice. Neurobiology of Aging, 31(1), 151–161. 10.1016/j.neurobiolaging.2008.03.002 18455269

[acel12958-bib-0003] Bergami, M. , Rimondini, R. , Santi, S. , Blum, R. , Götz, M. , & Canossa, M. (2008). Deletion of TrkB in adult progenitors alters newborn neuron integration into hippocampal circuits and increases anxiety‐like behavior. Proceedings of the National Academy of Sciences of the USA, 105(40), 15570–15575. 10.1073/pnas.0803702105 18832146 PMC2557028

[acel12958-bib-0004] Boldrini, M. , Fulmore, C. A. , Tartt, A. N. , Simeon, L. R. , Pavlova, I. , Poposka, V. , … Mann, J. J. (2018). Human hippocampal neurogenesis persists throughout aging. Cell Stem Cell, 22(4), 589–599.e5. 10.1016/j.stem.2018.03.015 29625071 PMC5957089

[acel12958-bib-0005] Bonaguidi, M. A. , Wheeler, M. A. , Shapiro, J. S. , Stadel, R. P. , Sun, G. J. , Ming, G. , & Song, H. (2011). In vivo clonal analysis reveals self‐renewing and multipotent adult neural stem cell characteristics. Cell, 145(7), 1142–1155. 10.1016/j.cell.2011.05.024 21664664 PMC3124562

[acel12958-bib-0006] Cameron, H. A. , & McKay, R. D. G. (1999). Restoring production of hippocampal neurons in old age. Nature Neuroscience, 2(10), 894–897. 10.1038/13197 10491610

[acel12958-bib-0007] Cipriani, S. , Ferrer, I. , Aronica, E. , Kovacs, G. G. , Verney, C. , Nardelli, J. , … Adle‐Biassette, H. (2018). Hippocampal radial glial subtypes and their neurogenic potential in human fetuses and healthy and Alzheimer’s disease adults. Cerebral Cortex, 28(7), 2458–2478. 10.1093/cercor/bhy096 29722804

[acel12958-bib-0008] Dennis, C. V. , Suh, L. S. , Rodriguez, M. L. , Kril, J. J. , & Sutherland, G. T. (2016). Human adult neurogenesis across the ages: An immunohistochemical study. Neuropathology and Applied Neurobiology, 42(7), 621–638. 10.1111/nan.12337 27424496 PMC5125837

[acel12958-bib-0009] Ekdahl, C. T. , Claasen, J.‐H. , Bonde, S. , Kokaia, Z. , & Lindvall, O. (2003). Inflammation is detrimental for neurogenesis in adult brain. Proceedings of the National Academy of Sciences of the USA, 100(23), 13632–13637. 10.1073/pnas.2234031100 14581618 PMC263865

[acel12958-bib-0010] Encinas, J. M. , Michurina, T. V. , Peunova, N. , Park, J.‐H. , Tordo, J. , Peterson, D. A. , … Enikolopov, G. (2011). Division‐coupled astrocytic differentiation and age‐related depletion of neural stem cells in the adult hippocampus. Cell Stem Cell, 8(5), 566–579. 10.1016/j.stem.2011.03.010 21549330 PMC3286186

[acel12958-bib-0011] Encinas, J. M. , Vaahtokari, A. , & Enikolopov, G. (2006). Fluoxetine targets early progenitor cells in the adult brain. Proceedings of the National Academy of Sciences of the USA, 103, 8233–8238. 16702546 10.1073/pnas.0601992103 PMC1461404

[acel12958-bib-0012] Eriksson, P. S. , Perfilieva, E. , Björk‐Eriksson, T. , Alborn, A.‐M. , Nordborg, C. , Peterson, D. A. , & Gage, F. H. (1998). Neurogenesis in the adult human hippocampus. Nature Medicine, 4(11), 1313–1317. 10.1038/3305 9809557

[acel12958-bib-0013] Farioli Vecchioli, S. , Sacchetti, S. , Nicolis di Robilant, V. , & Cutuli, D. (2018). The role of physical exercise and omega‐3 fatty acids in depressive illness in the elderly. Current Neuropharmacology, 16(3), 308–326. 10.2174/1570159X15666170912113852 28901279 PMC5843982

[acel12958-bib-0014] Ferreira, T. A. , Blackman, A. V. , Oyrer, J. , Jayabal, S. , Chung, A. J. , Watt, A. J. , … van Meyel, D. J. (2014). Neuronal morphometry directly from bitmap images. Nature Methods, 11(10), 982–984. 10.1038/nmeth.3125 25264773 PMC5271921

[acel12958-bib-0015] Filippov, V. , Kronenberg, G. , Pivneva, T. , Reuter, K. , Steiner, B. , Wang, L.‐P. , … Kempermann, G. (2003). Subpopulation of nestin‐expressing progenitor cells in the adult murine hippocampus shows electrophysiological and morphological characteristics of astrocytes. Molecular and Cellular Neurosciences, 23(3), 373–382. 10.1016/S1044-7431(03)00060-5 12837622

[acel12958-bib-0016] Gebara, E. , Bonaguidi, M. A. , Beckervordersandforth, R. , Sultan, S. , Udry, F. , Gijs, P.‐J. , … Toni, N. (2016). Heterogeneity of radial glia‐like cells in the adult hippocampus: Heterogeneity of RGL cells in the adult hippocampus. Stem Cells, 34(4), 997–1010. 10.1002/stem.2266 26729510 PMC5340291

[acel12958-bib-0017] Giachino, C. , Barz, M. , Tchorz, J. s. , Tome, M. , Gassmann, M. , Bischofberger, J. , … Taylor, V. (2014). GABA suppresses neurogenesis in the adult hippocampus through GABAB receptors. Development, 141(1), 83–90. 10.1242/dev.102608 24284211

[acel12958-bib-0018] Huttmann, K. , Sadgrove, M. , Wallraff, A. , Hinterkeuser, S. , Kirchhoff, F. , Steinhauser, C. , & Gray, W. P. (2003). Seizures preferentially stimulate proliferation of radial glia‐like astrocytes in the adult dentate gyrus: Functional and immunocytochemical analysis. European Journal of Neuroscience, 18(10), 2769–2778. 10.1111/j.1460-9568.2003.03002.x 14656326

[acel12958-bib-0019] Kaneko, N. , Kudo, K. , Mabuchi, T. , Takemoto, K. , Fujimaki, K. , Wati, H. , … Kanba, S. (2006). Suppression of cell proliferation by interferon‐alpha through interleukin‐1 production in adult rat dentate gyrus. Neuropsychopharmacology, 31(12), 2619–2626. 10.1038/sj.npp.1301137 16823390

[acel12958-bib-0020] Kempermann, G. , Jessberger, S. , Steiner, B. , & Kronenberg, G. (2004). Milestones of neuronal development in the adult hippocampus. Trends in Neurosciences, 27(8), 447–452. 10.1016/j.tins.2004.05.013 15271491

[acel12958-bib-0021] Knoth, R. , Singec, I. , Ditter, M. , Pantazis, G. , Capetian, P. , Meyer, R. P. , … Kempermann, G. (2010). Murine features of neurogenesis in the human hippocampus across the lifespan from 0 to 100 years. PLoS ONE, 5(1), e8809 10.1371/journal.pone.0008809 20126454 PMC2813284

[acel12958-bib-0022] Kohman, R. A. , Bhattacharya, T. K. , Kilby, C. , Bucko, P. , & Rhodes, J. S. (2013). Effects of minocycline on spatial learning, hippocampal neurogenesis and microglia in aged and adult mice. Behavioural Brain Research, 242, 17–24. 10.1016/j.bbr.2012.12.032 23274840 PMC3725815

[acel12958-bib-0023] Kuhn, H. , Dickinson‐Anson, H. , & Gage, F. (1996). Neurogenesis in the dentate gyrus of the adult rat: Age‐related decrease of neuronal progenitor proliferation. The Journal of Neuroscience, 16(6), 2027–2033. 10.1523/JNEUROSCI.16-06-02027 8604047 PMC6578509

[acel12958-bib-0024] Leuner, B. , Kozorovitskiy, Y. , Gross, C. G. , & Gould, E. (2007). Diminished adult neurogenesis in the marmoset brain precedes old age. Proceedings of the National Academy of Sciences of the USA, 104(43), 17169–17173. 10.1073/pnas.0708228104 17940008 PMC2040400

[acel12958-bib-0025] Lynch, M. A. (2010). Age‐related neuroinflammatory changes negatively impact on neuronal function. Frontiers in Aging Neuroscience, 1, 6 10.3389/neuro.24.006.2009 20552057 PMC2874409

[acel12958-bib-0026] Mignone, J. L. , Kukekov, V. , Chiang, A.‐S. , Steindler, D. , & Enikolopov, G. (2004). Neural stem and progenitor cells in nestin‐GFP transgenic mice. The Journal of Comparative Neurology, 469(3), 311–324. 10.1002/cne.10964 14730584

[acel12958-bib-0027] Mira, H. , Andreu, Z. , Suh, H. , Lie, D. C. , Jessberger, S. , Consiglio, A. , … Gage, F. H. (2010). Signaling through BMPR‐IA regulates quiescence and long‐term activity of neural stem cells in the adult hippocampus. Cell Stem Cell, 7(1), 78–89. 10.1016/j.stem.2010.04.016 20621052

[acel12958-bib-0028] Moreno-Jiménez, E. P. , Flor-García, M. , terreros-Roncal, J. , Rábano, A. , Cafini, F. , Pallas-bazarra, N. , … Llorens-Martín, M. (2019). Adult hippocampal neurogenesis is abundant in neurologically healthy subjects and drops sharply with Alzheimer´s disease. Nature Medicine. [Epub ahead of print]. 10.1038/s41591-019-0375-9 30911133

[acel12958-bib-0029] Ngwenya, L. B. , Heyworth, N. C. , Shwe, Y. , Moore, T. L. , & Rosene, D. L. (2015). Age‐related changes in dentate gyrus cell numbers, neurogenesis, and associations with cognitive impairments in the rhesus monkey. Frontiers in Systems Neuroscience, 9, 102 10.3389/fnsys.2015.00102 26236203 PMC4500920

[acel12958-bib-0030] Perera, T. D. , Dwork, A. J. , Keegan, K. A. , Thirumangalakudi, L. , Lipira, C. M. , Joyce, N. , … Coplan, J. D. (2011). Necessity of hippocampal neurogenesis for the therapeutic action of antidepressants in adult nonhuman primates. PLoS ONE, 6(4), e17600 10.1371/journal.pone.0017600 21525974 PMC3078107

[acel12958-bib-0031] Pilz, G.‐A. , Bottes, S. , Betizeau, M. , Jörg, D. J. , Carta, S. , Simons, B. D. , … Jessberger, S. (2018). Live imaging of neurogenesis in the adult mouse hippocampus. Science, 359(6376), 658–662. 10.1126/science.aao5056 29439238 PMC6986926

[acel12958-bib-0032] Santarelli, L. , Saxe, M. , Gross, C. , Surget, A. , Battaglia, F. , Dulawa, S. , Hen, R. (2003). Requirement of hippocampal neurogenesis for the behavioral effects of antidepressants. Science, 301(5634), 805–809. 10.1126/science.1083328 12907793

[acel12958-bib-0033] Saxe, M. d. , Battaglia, F. , Wang, J.‐w. , Malleret, G. , David, D. j. , Monckton, J. e. , … Drew, M. r. (2006). Ablation of hippocampal neurogenesis impairs contextual fear conditioning and synaptic plasticity in the dentate gyrus. Proceedings of the National Academy of Sciences of the USA, 103(46), 17501–17506. 10.1073/pnas.0607207103 17088541 PMC1859958

[acel12958-bib-0034] Segi‐Nishida, E. , Warner‐Schmidt, J. L. , & Duman, R. S. (2008). Electroconvulsive seizure and VEGF increase the proliferation of neural stem‐like cells in rat hippocampus. Proceedings of the National Academy of Sciences of the USA, 105(32), 11352–11357. 10.1073/pnas.0710858105 18682560 PMC2516270

[acel12958-bib-0035] Seri, B. , García‐Verdugo, J. M. , McEwen, B. S. , & Alvarez‐Buylla, A. (2001). Astrocytes give rise to new neurons in the adult Mammalian hippocampus. The Journal of Neuroscience, 21(18), 7153–7160. 10.1523/JNEUROSCI.21-18-07153.2001 11549726 PMC6762987

[acel12958-bib-0036] Sierra, A. , Martín‐Suárez, S. , Valcárcel‐Martín, R. , Pascual‐Brazo, J. , Aelvoet, S.‐A. , Abiega, O. , … Encinas, J. M. (2015). Neuronal hyperactivity accelerates depletion of neural stem cells and impairs hippocampal neurogenesis. Cell Stem Cell, 16(5), 488–503. 10.1016/j.stem.2015.04.003 25957904 PMC4443499

[acel12958-bib-0037] Snyder, J. S. , Soumier, A. , Brewer, M. , Pickel, J. , & Cameron, H. A. (2011). Adult hippocampal neurogenesis buffers stress responses and depressive behaviour. Nature, 476(7361), 458–461. 10.1038/nature10287 21814201 PMC3162077

[acel12958-bib-0038] Song, J. , Zhong, C. , Bonaguidi, M. A. , Sun, G. J. , Hsu, D. , Gu, Y. , … Song, H. (2012). Neuronal circuitry mechanism regulating adult quiescent neural stem‐cell fate decision. Nature, 489(7414), 150–154. 10.1038/nature11306 22842902 PMC3438284

[acel12958-bib-0039] Sorrells, S. F. , Paredes, M. F. , Cebrian‐Silla, A. , Sandoval, K. , Qi, D. , Kelley, K. W. , … Alvarez‐Buylla, A. (2018). Human hippocampal neurogenesis drops sharply in children to undetectable levels in adults. Nature, 555(7696), 377–381. 10.1038/nature25975 29513649 PMC6179355

[acel12958-bib-0040] Spalding, K. L. , Bergmann, O. , Alkass, K. , Bernard, S. , Salehpour, M. , Huttner, H. B. , … Frisén, J. (2013). Dynamics of hippocampal neurogenesis in adult humans. Cell, 153(6), 1219–1227. 10.1016/j.cell.2013.05.002 23746839 PMC4394608

[acel12958-bib-0041] van Praag, H. , Schinder, A. F. , Christie, B. R. , Toni, N. , Palmer, T. D. , & Gage, F. H. (2002). Functional neurogenesis in the adult hippocampus. Nature, 415(6875), 1030–1034. 10.1038/4151030a 11875571 PMC9284568

[acel12958-bib-0042] Walker, T. L. , Overall, R. W. , Vogler, S. , Sykes, A. M. , Ruhwald, S. , Lasse, D. , … Kempermann, G. (2016). Lysophosphatidic acid receptor is a functional marker of adult hippocampal precursor cells. Stem Cell Reports, 6(4), 552–565. 10.1016/j.stemcr.2016.03.002 27050949 PMC4834054

[acel12958-bib-0043] Walter, J. , Keiner, S. , Witte, O. W. , & Redecker, C. (2011). Age‐related effects on hippocampal precursor cell subpopulations and neurogenesis. Neurobiology of Aging, 32(10), 1906–1914. 10.1016/j.neurobiolaging.2009.11.011 20006411

[acel12958-bib-0044] Zhang, C.‐L. , Zou, Y. , He, W. , Gage, F. H. , & Evans, R. M. (2008). A role for adult TLX‐positive neural stem cells in learning and behaviour. Nature, 451(7181), 1004–1007. 10.1038/nature06562 18235445

[acel12958-bib-0045] Zheng, L.‐S. , Hitoshi, S. , Kaneko, N. , Takao, K. , Miyakawa, T. , Tanaka, Y. , … Sawamoto, K. (2014). Mechanisms for interferon‐α‐induced depression and neural stem cell dysfunction. Stem Cell Reports, 3(1), 73–84. 10.1016/j.stemcr.2014.05.015 25068123 PMC4110771

[acel12958-bib-0046] Ziebell, F. , Dehler, S. , Martin‐Villalba, A. , & Marciniak‐Czochra, A. (2018). Revealing age‐related changes of adult hippocampal neurogenesis using mathematical models. Development, 145(1), dev153544 10.1242/dev.153544 29229768 PMC5825879

