# Supplementary material for: Phenotypical and functional heterogeneity of neural stem cells in the aged hippocampus
Source: Aging Cell. 2019 Apr 15;18(4):e12958. doi: 10.1111/acel.12958 (PMC6612636; doi:10.1111/acel.12958)
Supplement: Supplementary file 1 [file ACEL-18-e12958-s001.pdf]

PHENOTYPICAL AND FUNCTIONAL HETEROGENEITY OF NEURAL STEM CELLS IN THE AGED HIPPOCAMPUS

SUPLEMENTARY INFORMATION

Extended Experimental Procedures

Animals

All the experiments were performed employing Nestin-GFP transgenic mice. All the animals were housed with ad libitum food and water access, in 12:12h light cycle. Nestin-GFP transgenic mice, kindly provided by Dr. Grigori Enikolopov at Cold Spring Harbor Laboratory (Cold Spring Harbor, NY, USA), were crossbred with C57BL/6 mice for at least 10 generations (Mignone et al. 2004).
All procedures were approved by the University of the Basque Country (EHU/UPV) Ethics Committees (Leioa, Spain) and the Comunidad Foral de Bizkaia (CEEA: M20/2015/236). All procedures followed the European directive 2010/63/UE and NIH guidelines. The mice were 3, 12 and 18 months old at the time of BrdU administration. The administration of IFN-α or saline started when the mice were 2 months old so that the animals were analyzed when they reached 3 months of age. The injection of KA or saline was performed in 12 months old animals. The number of animals used in each experiment was 4 for 3 months, 6 for 12 months and 3 for the 18 month point. For the experiment of KA 3 animals were used in each condition. For the experiment of IFN-α 5 animals were used for both conditions. For the short-term (1day) analysis and for the long-term (1 month) analysis 3 mice were used for vehicle and 4 for IFN-α. To analyze the effect of mynocycline 5 animals were used as a control and 4 for minocycline.

BrdU administration

BrdU (5-Bromo-1-(2-deoxy-β-D-ribofuranosyl)uracil, 5-Bromouracil deoxyriboside; Sigma, St Louis, MO, USA, REF: B5002) was diluted in sterile saline and administered through intraperitoneal injections at 150 mg/kg concentration. All the BrdU-injected mice were given four injections in the same day separated by 2-hr intervals. For all experiment the mice were sacrificed 24h after the last injection of BrdU.

Intrahippocampal injection of KA

12 months old mice were anesthetized with intraperitoneal ketamine (Ketolar, fizer)/xylazine (Sigma) (10:1 mg/kg) and received a single dose of the analgesic buprenorphine (1mg/kg) (Buprecare, Animalcare Lted) subcutaneously. After positioning in the stereotaxic apparatus, a 0.6mm hole was drilled at coordinates taken from Bregma: anteroposterior (AP) -1.8mm, laterolateral (LL) -1.6mm. A pooled glass microcapillary was inserted at -1.9mm dorsoventral (DV), and 50nL of saline (Sal), or KA (2-Carboxy-3-carboxymethyl-4-isopropenylpyrrolidine; Sigma-Aldrich, St Louis, MO, USA. REF: K0250) 0.74mM (Bouilleret et al. 1999), were delivered into the right hippocampus using a microinjector (Nanoject II, Drum mond Scientific, Broomal, PA, USA). After 2min, the microcapillary was retracted, and the mice sutured and maintained in a thermal blanket until recovered from anesthesia. The animals were monitored during the hours following the procedure.

IFN-α administration

IFN-α (Mintenyi Biotec, REF: 130-093-131) was diluted in sterile saline and administered intraperitoneally at $4x10^5$ IU/Kg. All mice received one single injection of IFN-α (at the same hour everyday), or saline, during 20 days and sacrificed 1day after the last injection in the short-term analysis or 1 month after the last injection of IFN in the long-term experiment.

Minocycline administration

Mynoclycline (Minocycline hydrochloride; Sigma, St Louis, MO, USA, REF: M9511) was diluted in the drinking drinking at a concentration of 0.533 mg/ml as mouse drank 6 ml within a day they would receive a dose of 100 mg/kg/day. Control mice received normal water without any addition.

Mice received minocycline in their drinking water for a total of 30 days. The amount of liquid intake was measured daily.

Immunohistochemistry

Experiments were performed essentially as described before following methods optimized for the use in transgenic mice (Encinas et al. 2006; Encinas and Enikolopov 2008; Encinas et al. 2011). Animals were deeply anesthetized and were subjected to transcardial perfusion with 30 ml of PBS followed by 30 ml of 4% (w/v) paraformaldehyde in PBS, pH 7.4. The brains were removed, cut longitudinally into two hemispheres and postfixed, with the same fixative, for 3 hr at room temperature, then transferred to PBS and kept at 4°C. Serial 50 µm-thick sagittal sections were cut using a Leica VT 1200S vibrating blade microtome (Leica Microsystems GmbH, Wetzlar, Germany). Immunostaining was carried out following a standard procedure: the sections were incubated with blocking and permeabilization solution (PBS containing 0.25% Triton-100X and 3% BSA) for 3hr at room temperature, and then incubated overnight with the primary antibodies (diluted in the same solution) at 4°C. After thorough washing with PBS, the sections were incubated with fluorochrome-conjugated secondary antibodies diluted in the blocking and permeabilization solution for 3 hr at room temperature. After washing with PBS, the sections were mounted on gelatin coated slides with DakoCytomation Fluorescent Mounting Medium (DakoCytomation, Carpinteria, CA). Those sections destined to the analysis of BrdU incorporation were treated, before the immunostaining procedure, with 2N HCl for 20 min at 37°C, rinsed with PBS, incubated with 0.1M sodium tetraborate for 10 min at room temperature, and then rinsed with PBS. The GFP signal from the transgenic mice was detected with an antibody against GFP for enhancement and better visualization. The following antibodies were used: chicken anti-GFP (Aves Laboratories, Tigard, OR) at 1:1000 dilution; rabbit anti-Ki67 (Vector Laboratories, Burlingame,CA, USA) at 1:1000; rabbit anti-GFAP (Dako Cytomation) at 1:1000; rabbit anti-S100β (DakoCytomation) at 1:500; rat anti-BrdU (AbD Serotech, Kidlington, UK) at 1: 400; AlexaFluor 488 goat anti-chicken (Molecular Probes, Willow Creek Road, Eugene, OR) at 1:500; AlexaFluor 647 goat anti-rabbit (Molecular Probes) at 1:500; AlexaFluor 568 goat anti-rat (Molecular Probes) at 1:500; DAPI, at 1:1000 (Sigma) was used at counterstaining when required.

Image capture

All fluorescence immunostaining images were collected employing a Leica SP8 (Leica, Wetzlar, Germany) laser scanning microscopes and their corresponding manufacturer's software. The signal from each fluorochrome was collected sequentially, and controls with sections stained with single fluorochromes were performed to confirm the absence of signal leaking into different channels and antibody penetration. All images were imported into Adobe Photoshop 7.0 (Adobe Systems Incorporated, San Jose, CA) in tiff format. Brightness, contrast, and background were adjusted equally for the entire image using the "brightness and contrast" and "levels" controls from the "image/adjustment" set of options without any further modification. Al images shown are projections from z-stacks ranging from 10 (typically for individual cell images) to 20 microns of thickness.

Cell quantification

Quantitative analysis of cell populations (proliferation) in Nestin-GFP mice was performed by design-based (assumption free, unbiased) stereology using a modified optical fractionator sampling scheme as previously described (Encinas et al. 2004; Encinas and Enikolopov 2008; Encinas et al. 2011). Slices were collected using systematic-random sampling. The hemisphere was sliced sagittally in a lateral-to-medial direction, from the beginning of the lateral ventricle to the middle line, thus including the entire DG. The 50 µm slices were collected in 5 parallel sets, each set consisting of 14 slices, each slice 300 µm apart from the next. All BrdU cells per slice were counted, with a 63x oil immersion objective, to obtain absolute numbers of BrdU cells. α-NSCs were defined as radial glia-like cells positive for Nestin-GFP and GFAP with the soma located in the SGZ or the lower third of the GCL and with a process extending from the SGZ towards the molecular layer through the GCL. Ω-NSCs were Nestin-GFP and GFAP positive with a multibranches phenotype and the soma placed out of SGZ. The relative proportions of BrdU-

positive NSCs types were referred to the total number of NSCs quantified per animal. For the absolute number of NSCs, the number of NSCs, excluding those in the uppermost focal plane, was counted in 100 µm-wide, 50 µm-tall 12 µm-deep z-stacks, in GFP and GFAP stained slices from Nestin-GFP mice, using a 63x oil immersion objective. At least 4 z-stacks were obtained from each slice. The values were normalized to the total volume of the SGZ+GCL for each animal. The total volume was obtained by measuring the area of the SGZ+GCL in each slice and measuring the thickness of each slice in at least 3 points. To measure the morphological changes in NSCs, at least 50 cells were randomly selected form 20 µm-thick z-stacks taken form Sal, LKA, IFN-α mice brain sections immunostained for GFP and GFAP. Primary processes were considered to be those emerging directly from the soma, and the secondary processes those emerging from the primary processes in the 30 µm closest to the soma.The area of DG in each z-stack was quantified using the Fiji Is Just ImageJ (Fiji) distribution of ImageJ, Using the total hippocampal volume, estimated in low magnification images of the whole series taken in a confocal microscope using Las AF lite.

Sholl analysis

Sholl analysis is an open-source plug-in for FIJI (Image J, Schindelin et al.2012) this plug-in performs the Sholl technique directly on 2D or 3D images of fluorescence labeled cells. It is based on an algorithm to retrieve data from pixel-based connectivity (detailed in the user guide of http://fiji.sc/Sholl). For the morphological analysis of NSCs we obtained 3D reconstructions from confocal stack images(1024 pixels of resolution) . Single NSCs were analyzed using 3D Sholl analysis plugin (http://fiji.sc/Sholl_Analysis) as described in (Ferreira et al., 2014).

Z-stack from Nestin-GFP/GFAP positive NSCs were collected in a random manner.Only entire NSCs were choosen to analize the complexity of the cells in the analisys. The thickness of each z-stack depends on the NSCs, as the image must include all the cell body and the all the volumen ocupied by the branches and arborizations. Using the tool “polygon” of the Image J the outline of the celsl was delimited. Next, using the threshold we elaborate a mask to remove the background from the delimited image obtaining a single image which corresponds with our NSCs. Finally, using the tool “line” we draw a line from the middle of the some to the farthest arborization or segment of the NSC to stablish the lenght of the NSCs and the limit for the analysis. At least 25 cells form each 3 m.o. mice and 13 from each 12 m.o. and 18 m.o. mice were analyzed. For the analysis of IFN at least 20 cells were analyzed in the short-term experiments and at least 40 in the long-term. At least 50 cells were analyzed in the minocycline experiment. Images were captured in systemazied random manner. A field of vision (at 63x magnification) was placed in the tip of upper blade of the GCL and images were taken skipping the folloging 3 fields and moving along the GCL towards the medial portion and continuing in the lower blade (in a lateral direction). Images were taken only when the cell body and the arborization were totally inside the slice

Alpha- and omega-cell classification model.

The classification method utilizes an equation obtained after performing a stepwise selection of Sholl analysis related variables based on their correlation matrix and principal component analysis, and subsequent generation of a logistic regression model with a batch of classified cells. The model was validated using a batch of manually classified cells not used for the training. Different variables obtained from Sholl analysis were analyzed using a correlation matrix to eliminate those variables with very high and significant correlation indexes with at least one of the other variables. Then, a principal component analysis was performed using the selected variables and used to select those variables which showed the greatest influence in the variance of the data. Finally, a logistic regression model for the selected Sholl variables was fitted using a subset of classified data, and the remaining data used as a test to validate the model. Correlation matrix and principal component analysis were performed using the Past3 software (Hammer, Ø, Harper, D.A.T., and P. D. Ryan, 2001. PAST: Paleontological Statistics Software Package for Education and Data Analysis. Palaeontologia Electronica 4(1): 9pp.) plus the logistic regression model using JASP (JASP Team (2018). JASP (Version 0.9)[Computer software]). The macro is available for download here:https://www.achucarro.org/downloads.

Statistical analysis.

SigmaPlot (San Jose, CA, USA) was used for statistical analysis. 1-way ANOVA test was performed to determine the effect of the factor (Figure 1-3). In all cases, all pair-wise multiple comparisons (Holm-Sidak method or Dunn´s) were set as a post-hoc test to determine the significance between groups in each factor. For analysis of pairs of groups (Figure 4-6), a Student´s t test was performed. Hierarchical clustering was performed using Ward's method and squared Euclidean distances as linkage metric. For the Sholl analysis two-way repeated measures ANOVA followed by Bonferroni post-hoc test was performed. Only $p<0.05$ is reported to be significant. Data are shown as mean ± SEM (standard error of the mean). For the IFN-α experiment 2-way ANOVA was performed to analyzed the interaction between factors (age x treatment), no interactions were found. The fitting of linear and nonlinear regression models to data was compared in GraphPad Prism 5 (GraphPad Software, Incl, San Diego, CA) using Akaike's information criterion with correction for finite sample sizes (AICc) (Hurvich and Tsai, 1993). The exponential growth equation showed the best fit based on AICc with a normal distribution of the residuals, analyzed using the Shapiro Wilk normality test. In addition, correlation of these data was analyzed using the Pearson correlation coefficient.

Extended Experimental Procedures References

1. Mignone JL, Kukekov V, Chiang AS, Steindler D, & Enikolopov G (2004) Neural stem and progenitor cells in nestin-GFP transgenic mice. *J Comp Neurol* 469(3):311-324.

2. Bouilleret V, Ridoux V, Depaulis A, Marescaux C, Nehlig A, Le Gal La Salle G.(1999) Recurrent seizures and hippocampal sclerosis following intra-hippocampal kainate injection in adult mice: electroencephalography, histopathology and synaptic reorganization similar to mesial temporal lobe epilepsy. *Neuroscience.*89:717–729.

3. Encinas JM, Vaahtokari A, & Enikolopov G (2006) Fluoxetine targets early progenitor cells in the adult brain. *Proc Natl Acad Sci U S A* 103(21):8233-8238.

4. Encinas JM and Enikolopov G (2008) Identifying and quantitating neural stem and progenitor cells in the adult brain.*Method Cell Biol*. 85: 243-72.

5. Encinas JM*, et al.* (2011) Division-coupled astrocytic differentiation and age-related depletion of neural stem cells in the adult hippocampus. *Cell Stem Cell* 8(5):566-579.

6. Schindelin J, Arganda-Carreras I, Frise E, Kaynig V, Longair M, Pietzsch T, Preibisch S, Rueden C, Saalfeld S, Schmid B, Tinevez JY, White DJ, Hartenstein V, Eliceiri K, Tomancak P, Cardona A.(2012) Fiji: an open-source platform for biological-image analysis. *Nat Methods*. Jun 28;9(7):676-82.

7. Ferreira T, Blackman A, Oyrer J, Jayabal A, Chung A, Watt A, Sjöström J, van Meyel D.(2014) Neuronal morphometry directly from bitmap images, *Nature Methods* 11(10): 982–984.

8. Hurvich, C.M., Tsai, C.L., (1993). A corrected Akaike information criterion for vector autoregressive model selection. *Journal of Time Series* 14, 271–279.

Supplemental Figures

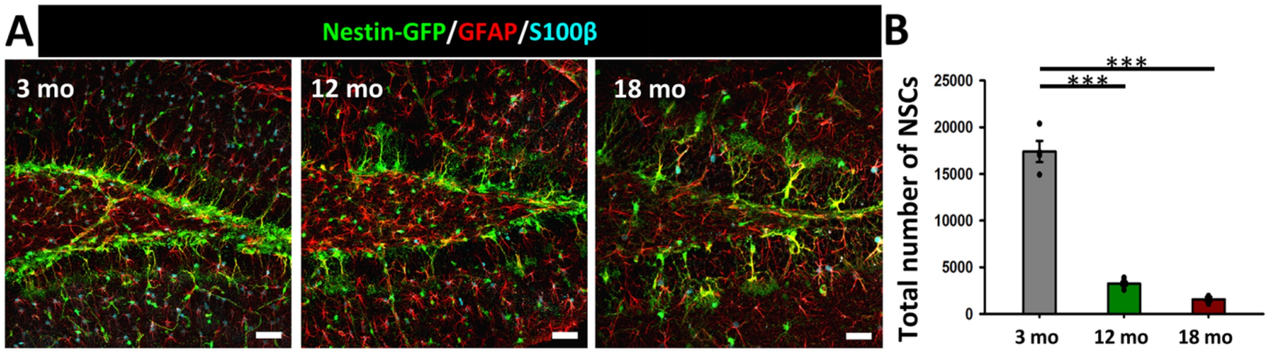

Suplementary Information Figure 1. The population of NSCs and dividing NSCs decrease with age. Confocal microscopy images showing total population of NSCs (Nestin-GFP-positive, GFAP immunopositive but S100 β-negative cells) located in the SGZ at 3 (left), 12 (middle) and 18 (right) m.o. mice. **(B)** Quantification of total number of NSCs (Nestin-GFP-positive, GFAP-immunopositive cells) in the SGZ showing the significant decrease of the NSCs population with age. (***p < 0.001 One way ANOVA Holm Sidak post hoc test. Bars show mean ± SEM. Scale bar is 20 μm. Dots show individual data.

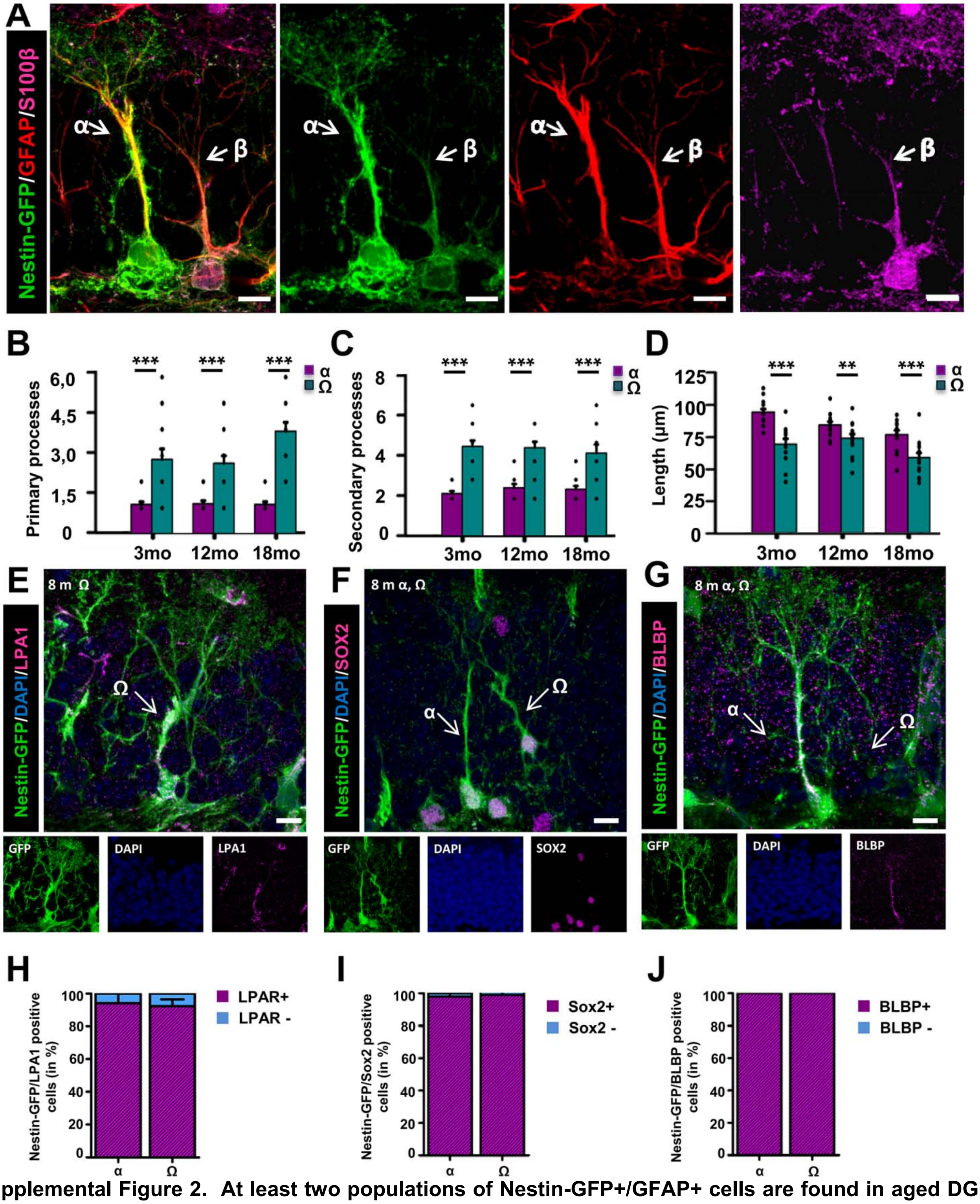

Supplemental Figure 2. At least two populations of Nestin-GFP+/GFAP+ cells are found in aged DG. (A) Confocal microscopy image of α-cell and β-cell, β-cell are immunopositive to Nestin-GFP, GFAP and S100 β in clearly contrast to α-cell which does not express S100 β. (B) Quantification of the number of primary processes, in α-cells and a Ω-cells, defined as those emerging from the soma, in 3, 12 and 18 months old mice, showing a significant increase in a Ω-cells regardless the age. (C) Quantification of the number of secondary processes, defined as those branching from the primary process primary processes, showing a significant increase in a Ω-cells in all timepoint. (D) Quantification of the length (center of soma to furthest tip of NSCs) showing a significant decrease in Ω-cells in all ages. (E) Confocal microscopy image showing the expression of LPA in Ω-cells. (F) Confocal microscopy image showing the expression of Sox2 in α-cell and Ω-cell (G) Confocal microscopy image showing the expression of BLBP in Ω-cells. (H) Quantification of the expression of LPA1 by α-cells and Ω -cells in percentage. (I) Quantification of the expression Sox2 by α-cells and Ω-cells in percentage. (J) Quantification of the expression of BLBP by α-cells and Ω-cells in percentage. Scale bar is 10 μm in a, e- g **$p < 0.01$, ***$p < 0.001$ after all pairwise multiple comparisons by Holm-Sidak post hoc test. Bars show mean ± SEM. Dots show individual data.

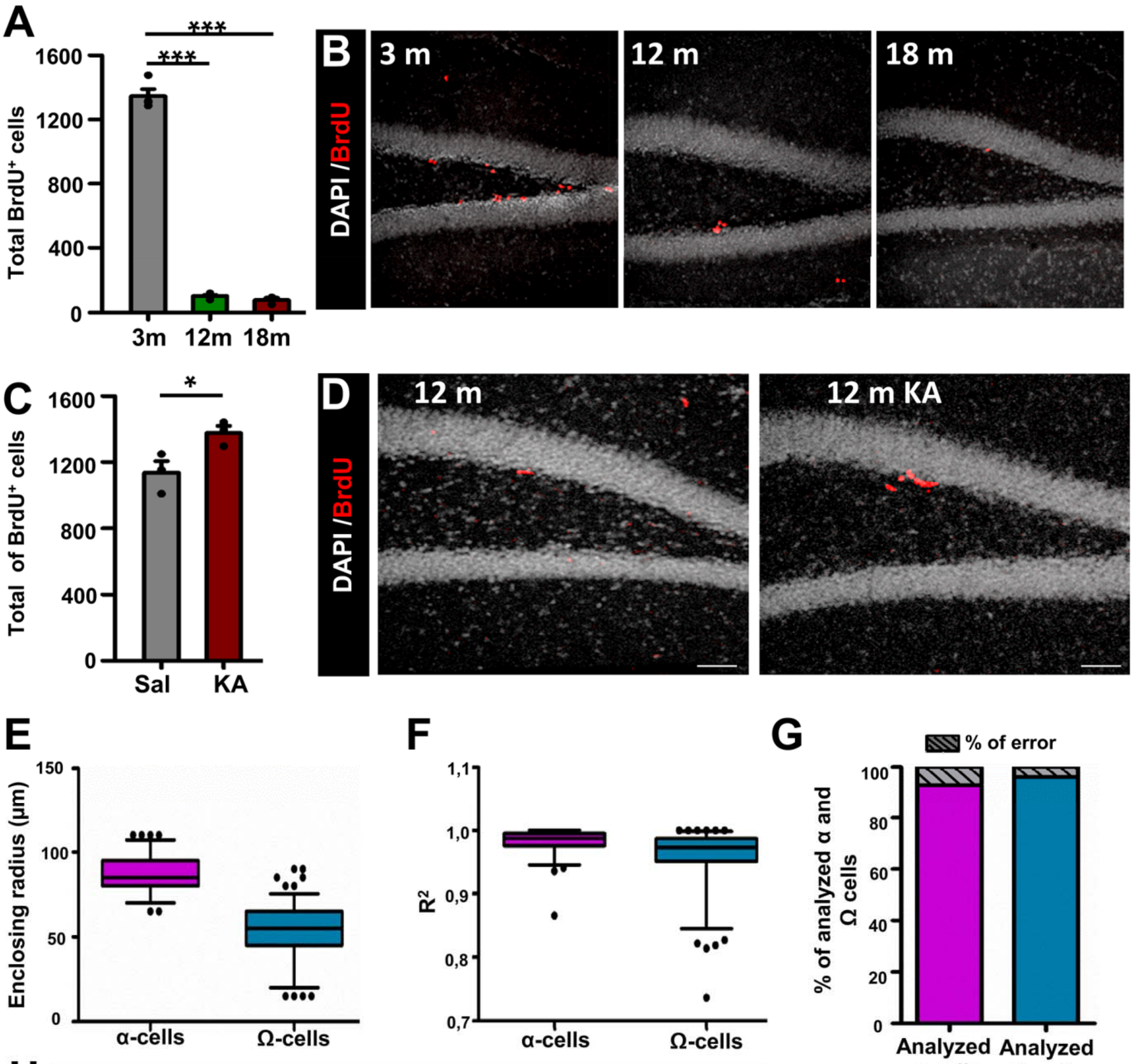

H

Parameter	Estimate	Standard Error	z	p
Intercept	153.623	57.853	2.655	0.008
Enclossing radius	-0.496	0.163	-3.041	0.002
50 microns Coefficient of determination (log-log)	-119.269	49.814	-2.394	0.017

Supplemental Figure 3. Ω-cells divide with lower probability even in pro-activation conditions. (A) Quantification of total number of dividing cells (BrdU immunopositive cells) in the SGZ showing the significant decrease of the dividing cells with age. ***p < 0.001 One way ANOVA Holm Sidak post hoc test. Bars show mean ± SEM. Scale bar is 50 µm. Dots show individual data. (B) Confocal microscopy images showing total BrdU positive cells located in the SGZ at 3 (left), 12 (middle) and 18 (right) m.o. mice. (C) Quantification of total number of dividing cells (BrdU immunopositive cells) in the SGZ of 12 m.o. and Kainate injected 12 m.o mice, showing the significant increase of the dividing cells in Kainate injected mice.(D) Zoomed out images showing the increase in total number of BrdU positive cells in kainate treated mice. (E) The variable “enclosing radius” is the widest of intersecting and represents the maximum distance between the center of the soma and the border of the dedritic tree. (F) The variable “50 µm log-log R^2” is the coefficient of determination of the log-log regression of a Sholl analysis (intersections vs radii) performed within a maximum radius of 50 µm. (G) Quantification of the accuracy of the model used to classify NSCs. (H) Values corresponding to each parameter in the logistic regression model. ***p < 0.001, One way ANOVA Holm Sidak post hoc test. *p < 0.05 by Student’s t test. Mann-Whitney test. Whiskers in box plots represent 5th and 95th percentiles, and the line the 50th percentile. Bars show mean ± SEM. Dots show individual data. Scale bar is 50 µm in B and D..

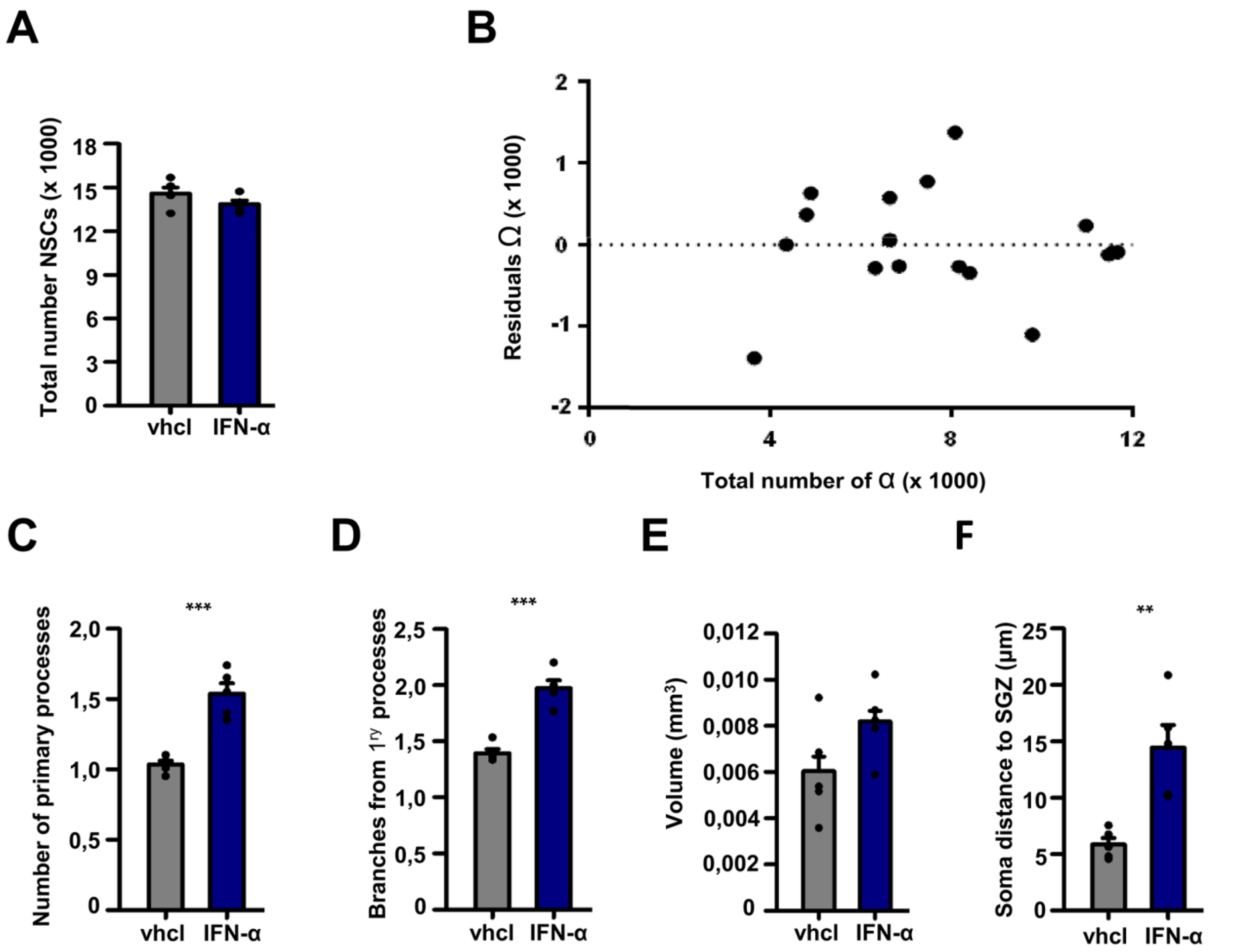

Supplemental Figure 3. Chronic inflammation increase the complexity of NSCs. (A) Total number of NSCs (α-cells + Ω-cells). (B) Residuals derived from the regression analysis confirming the adequacy of the model. (C) Quantification of the number of NSC primary processes, defined as those emerging from the soma, IFN-α injected mice presented more number of primery processes. (D) The number of NSC secondary processes, defined as those branching from the primary process, is also increased in injected mice. (E) Quantification of the cell volume showing the increase of the volume occupied by NSCs in IFN-α treated mice. (F) Quantification of the distance between the center of cell body and the SGZ. The soma of the IFN-α treated NSCs moved up into SGZ. $^{**}p < 0.01$, $^{***}p < 0.001$ by Student's t test. Bars show mean ± SEM. Dots show individual data
